# Methanol as a co-substrate with CO_2_ enhances butyrate production in microbial electrosynthesis

**DOI:** 10.1007/s00253-024-13218-y

**Published:** 2024-06-14

**Authors:** Hui Yao, Johanna M. Rinta-Kanto, Igor Vassilev, Marika Kokko

**Affiliations:** Faculty of Engineering and Natural Sciences, Tampere University, Korkeakoulunkatu 8, 33720 Tampere, Finland

**Keywords:** Methanol utilisation, CO_2_ utilisation, Microbial electrosynthesis, Butyrate, Electron donor

## Abstract

**Abstract:**

Methanol is a promising feedstock for the bio-based economy as it can be derived from organic waste streams or produced electrochemically from CO_2_. Acetate production from CO_2_ in microbial electrosynthesis (MES) has been widely studied, while more valuable compounds such as butyrate are currently attracting attention. In this study, methanol was used as a co-substrate with CO_2_ to enhance butyrate production in MES. Feeding with CO_2_ and methanol resulted in the highest butyrate production rates and titres of 0.36 ± 0.01 g L^−1^ d^−1^ and 8.6 ± 0.2 g L^−1^, respectively, outperforming reactors with only CO_2_ feeding (0.20 ± 0.03 g L^−1^ d^−1^ and 5.2 ± 0.1 g L^−1^, respectively). Methanol acted as electron donor and as carbon source, both of which contributed ca. 50% of the carbon in the products. *Eubacterium* was the dominant genus with 52.6 ± 2.5% relative abundance. Thus, we demonstrate attractive route for the use of the C1 substrates, CO_2_ and methanol, to produce mainly butyrate.

**Key points:**

• *Butyrate was the main product from methanol and CO*_*2*_ *in MES*

• *Methanol acted as both carbon and electron source in MES*

• *Eubacterium dominating microbial culture was enriched in MES*

**Graphical Abstract:**

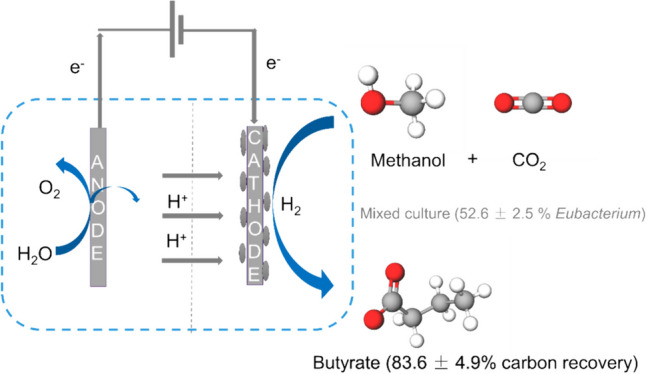

**Supplementary information:**

The online version contains supplementary material available at 10.1007/s00253-024-13218-y.

## Introduction

In light of replacing fossil fuel-based chemicals with renewable chemicals and reaching the goal of a carbon neutral society, it is crucial to explore new routes for the production of renewable biochemicals. The greenhouse gas CO_**2**_ is considered as the major cause of the current climate warming, while it can also be regarded as an alternative carbon feedstock for the production of organic commodities (Aresta and Dibenedetto 2007). Such CO_**2**_ conversion can be realised with microbial electrosynthesis (MES) where microorganisms are employed as biocatalysts to reduce CO_**2**_ to multi-carbon compounds with an external energy source. In MES, an electrochemical cell is used to provide electrons for microorganisms. In the anode, water is oxidised to release oxygen, electrons, and protons. Electrons are transferred through external load to the cathode, and the protons migrate to the cathode via ion exchange membrane. At the cathode, the protons can be abiotically/biotically reduced to H_2_ that can be used for CO_2_ reduction, or alternatively, CO_2_ reduction can be achieved by microorganisms with direct utilization of electrons from the cathode electrode (Rabaey and Rozendal 2010). Since the first proof of concept of MES (Nevin et al. 2010), various studies have been undertaken during the past decade to improve the performance of MES from different perspectives, such as improvement of electrode materials (Sharma et al. 2019), reactor configuration design (Vassilev et al. 2019), and optimisation of operation parameters (Marshall et al. 2013). At the early stage, the main product of MES was acetate, which possesses limited economic value and thus, weakens the competitiveness of MES for the synthesis of chemical commodities. In order to expand the product spectrum of MES, enriching a certain microbiome (Vassilev et al. 2018) or optimising operational parameters (Jourdin et al. 2019) have been studied resulting in the production of longer chain fatty acids such as butyrate, iso-butyrate, caproate, and the corresponding alcohols.

Butyrate, as a widely used compound in industries (e.g. food, chemical, and pharmaceutical industry), possesses a higher economic value than acetate. Butyrate production in MES has been demonstrated since 2015 and has been accelerated by adding a chemical electron donor (e.g. ethanol) to reach butyrate titres of 6.3 g L^−1^ (Ganigué et al. 2015; Izadi et al. 2021b; Li et al. 2022). However, ethanol is a widely used biofuel, and the current industrial production of ethanol mainly relies on agricultural products, e.g. sugarcane and corn (Medina and Magalhaes 2021). The competition with food production makes ethanol a less promising substrate for MES from a sustainability point of view (Aro 2016). Therefore, seeking more sustainable electron donors with sufficient reducing power could improve the production of higher-value products in MES.

Methanol is a promising feedstock for bioproduction. About 80% of the methanol used in the world is currently produced from petroleum products (synthesis gas) at elevated pressure (50–100 bar) and temperature (200 to 300 ℃) (Iaquaniello et al. 2017). Besides, sustainable production processes of methanol have been developed recently, using biomass or organic waste streams as substrates, which has been implemented at the industrial level by using municipal waste, including unrecyclable plastics (Hobson and Márquez 2018). In addition to its sustainable production, methanol is also abundant in certain industrial waste streams, e.g. in pulp and paper industry (Mäki et al. 2021). The methanol-rich waste streams can be directly used as the feedstock for the bioproduction of value-added products (Eregowda et al. 2021).

Methanol can act as the carbon and energy source to support the growth of microorganisms, including acetogens (Ginige et al. 2009). Acetogens are autotrophic bacteria utilising C1 compounds through the Wood-Ljungdahl pathway (WLP) with acetate as the major product. WLP is considered as one of the most energetically efficient non-photosynthetic carbon fixation pathways, and therefore acetogens have gained attention to set a carboxylate platform for the sustainable biochemical synthesis. It has been suggested that methanol could also be utilised through the WLP (Bache and Pfennig 1981), similar to the utilisation of other C1 compounds, such as CO_2_ (Litty et al. 2022), CO (Diender et al. 2015), and formate (Balch et al. 1977). Methanol is a promising reducing agent for microorganisms, which can facilitate the carbon chain elongation of shorter chain fatty acids (acetate or propionate) to longer chain fatty acids, including n-butyrate, i-butyrate, valerate, and caproate (Chen et al. 2017, 2020; De Leeuw et al. 2020). Although the fundamentals of methanol carbon chain elongation are not fully understood, methanol has been suggested to provide reducing power to drive the carbon chain elongation via different pathways, one of which is the reverse beta oxidation (De Smit et al. 2019). The two-carbon acetyl-coenzyme A (acetyl-CoA) is considered as the chain elongation block molecule, extending the carbon chain by two carbons at each time, i.e. acetate (C2) to butyrate (C4) or propionate (C3) to valerate (C5) (Kallscheuer et al. 2017). In MES, an electron donor (such as ethanol) addition has been proven to provide reducing power and result in the production of more reduced products than acetate (Izadi et al. 2021b). Thus, methanol also possesses the potential to provide extra reducing power in MES, while to the authors’ knowledge, it has not been reported.

In this study, the use of methanol as co-substrate in MES was investigated due to its potential role as a carbon source and reducing agent. The feasibility and role of utilising methanol as the co-substrate with CO_2_ in MES was investigated by feeding MES with either methanol or CO_2_ or with a combination of both compounds. MES performance was evaluated by analysing the product formation, substrate utilisation, carbon and electron balances as well as by characterising the microbiomes.

## Materials and methods

### MES reactor setup

Both abiotic and biotic experiments were performed in double chamber flat plate reactors. The reactor comprised acrylic rectangular plates to create two compartments, each with an internal volume of 35 mL (7 cm × 5 cm × 1 cm) (Fig. S1). Cation exchange membrane (CMI-7000, Membranes International, USA) with a surface area of 35 cm^2^ was used to separate the anodic and cathodic chambers. In the cathodic chamber, graphite granules (10 ± 2 mm, EC-100, Graphite Sales Inc, USA) were used to fill the compartment, serving as the cathode electrode and carrier for microbial growth. Two graphite rods (diameter 6 mm, length 15 cm, Sigma-Aldrich, USA) were placed in the packed granular bed and connected to the external circuit as current collectors. An Ag/AgCl reference electrode (+ 0.206 V vs. normal hydrogen electrode, BASi, USA) was connected to a capillary glass frit (4 × 60 mm, Prosense, the Netherlands) immersed in the cathodic chamber. In the anodic chamber, a platinum wire (0.4 mm, 99.95%, Advent research materials, UK) worked as the counter electrode. Catholyte and anolyte (300 mL of 50 mmol L^−1^ H_2_SO_4_) were recirculated through external bottles with a peristaltic pump at a flow rate of 40 mL min^−1^. Gases from the cathodic recirculation bottle were collected in a 5 L gas bag (Supel™-Inert Gas Sampling Bags, MERCK, Germany). The system was connected to a potentiostat (VMP3, BioLogic, France), and all the potentials are reported with respect to the Ag/AgCl reference electrode. All the experiments were run at 35 ℃.

### Medium composition and microbial culture enrichment

The medium used in the cathodic chamber consisted of 18 g L^**−**1^ Na_2_HPO_4_·2H_2_O, 3 g L^**−**1^ KH_2_PO_4_, 3 g L^**−**1^ NH_4_Cl, 15 mg L^**−**1^ CaCl_2_, 20 mg L^**−**1^ MgSO_4_·7H_2_O, 2.1 g L^**−**1^ sodium 2-bromoethanesulfonate, 1 g L^**−**1^ yeast extract as well as 10 mL L^**−**1^ trace elements solution and 1 mL L^**−**1^ vitamin solution (Table S1 and S2). The medium had a pH of ca. 7.2, and it was sterilised by filtration (0.2 µm, Fisherbrand™ Disposable PES Filter Units, USA) before use. The medium was sparged with N_2_ for 30 min to remove oxygen before starting experiments.

The original inoculum was a mixed culture originating from cow rumen and enriched for carboxylate production in an MES reactor, which was fed with CO_2_ and current over a period of one year. Microbial cultures were further enriched in two parallel MES reactors. The reactor setup was similar to the one described above, except the internal volume of each chamber was 124 mL and the projected surface area of the membrane was 41 cm^2^. A total of 600 mL of medium and 80 mL of inoculum were added as the catholyte to start the enrichment. A total of 300 mL of 50 mmol L^**−**1^ H_2_SO_4_ (prepared in milli-Q water) was added as the anolyte and replenished every two weeks during the whole operation. In this study, both anolyte and catholyte were recirculated with the rate of 40 mL min^**−**1^. CO_2_ was sparged to the cathodic recirculation bottle every 2 to 4 days for 15 min at the rate of 0.4 L min^**−**1^ (EK-2LR rotameter, Kytola, Finland). After CO_2_ sparging, methanol stock solution (1.2 mol L^**−**1^, prepared in medium) was added to reach a methanol concentration of 20 mmol L^**−**1^ in the catholyte. The pH of the catholyte was measured after CO_2_ and methanol addition. If necessary, 1 mol L^**−**1^ NaOH was added to maintain the catholyte pH between 6.0 and 6.2. The enrichment reactors were operated in semi-batch mode, and the medium in the cathodic recirculation bottles (the bottle was emptied and 500 mL of fresh medium was added) was replaced every 35 to 50 days to provide microorganisms with fresh nutrients. In total, the enrichment of microbial culture was operated for over 240 days (five semi-batches in total), during which the cathodic potential was controlled at a range of − 0.75 to − 0.95 V.

### Determining the role of methanol in MES

To understand the role of methanol as a co-substrate in MES, three different substrate feedings were tested: carbon dioxide (CD), methanol (ME), and co-feeding of carbon dioxide and methanol (ME-CD). Two replicates were operated with each substrate feeding. In the beginning, 330 mL of medium and 150 mL of H_2_SO_4_ were added as catholyte and anolyte, respectively. The reactors were inoculated with 40 mL of cathodic broth from the enrichment reactors and operated for 30–32 days. The cathodic current was controlled at − 100 mA, and the cathodic potential was recorded. Substrate(s) were added three times a week by following the same procedure as with the enrichment reactors. The detailed operational parameters are shown in Table 1.

**Table 1:** Operational parameters of the enrichment and co-substrate experiments

Experiment	Codes	Substrate(s) addition
Enrichment	R	CO_2_ (15 min, 0.4 min L^−1^) Methanol (20 mmol L^−1^)
Co-substrate	ME-CD	CO_2_ (15 min, 0.4 min L^−1^) Methanol (20 mmol L^−1^)
	ME	Methanol (20 mmol L^−1^)
	CD	CO_2_ (15 min, 0.4 min L^−1^)

The potential abiotic methanol removal, i.e. electrochemical reduction of methanol or diffusion of methanol through the membrane, was examined in abiotic conditions. The abiotic experiment was carried out for 7 days with an identical MES reactor setup and with the same operating parameters as in the co-substrate experiment. The connecting tubing and graphite granules were autoclaved before use. The reactor plates were sterilised with 70% ethanol and assembled in a laminar flow hood. Yeast extract and vitamin solution were removed from the medium to minimise the possibility of microbial growth during the abiotic experiment. CO_2_ and methanol were added according to Table 1, and samples were taken on days 0, 2, 4, and 7 from both the anode (liquid samples) and the cathode (liquid and gas samples).

### Analytical methods and calculations

Liquid samples were taken before and after the CO_2_ and/or methanol addition from the catholyte recirculation bottles (three times a week). Catholyte pH was immediately measured with a pH meter (WTW-330i, Germany) and optical density (OD at 600 nm) with a UV–Vis spectrophotometer (UV-1800, Shimadzu, Japan). The remaining liquid samples were filtered through a 0.2 μm filter (CHROMAFIL Xtra PET, 25 mm, 0.2 µm, MACHERREY-NAGEL, Germany) and used for analysing total inorganic carbon (TIC) via a total organic carbon analyser with ASI-V sampler (TOC-VCPH, Shimadzu, Japan). Sample pH was firstly adjusted to below 3 using 3 mol L^**−**1^ HCl to volatilise the inorganic carbon into CO_2_, which was detected by a nondispersive infrared sensor.

Volatile fatty acids (VFAs) and alcohols (i.e. methanol, ethanol, butanol, acetate, propionate, butyrate, isobutyrate, valerate, caproate) were analysed via gas chromatography with a flame ionisation detector (GC-FID 2010 Plus, Shimadzu, Japan), equipped with AOC-20s autosampler and a Zebron ZB-WAX plus column (0.25 µm diameter, 30 m length). Helium was used as the carrier gas at a flow rate of 84.4 mL min^**−**1^ and pressure of 114.6 kPa. The column temperature was firstly set at 40 ℃ and held for 2 min, followed by the heating-up program to 160 ℃ with a rate of 20 ℃ min^**−**1^, and further increase the temperature to 220 ℃ with a rate of 40 ℃ min^**−**1^, then hold for 3 min. The injector and detector temperatures were set at 250 ℃. The repeated measures ANOVA was carried out to analyse the differences of VFA productions between ME-CD and CD groups.

Headspace gas composition (i.e. H_2_, CO_2_, CH_4_) was analysed via gas chromatography with a thermal conductivity detector (GC-TCD 2014, Shimadzu, Japan) equipped with an Agilent J&W packed GC column (1.8 m length, 2 mm Porapak, the Netherlands). N_2_ was used as the carrier gas at a flow rate of 20 mL min^**−**1^. The temperature of the injector, column, and detector was set at 110 ℃, 80 ℃, and 110 ℃, respectively. The volume in the gas bags was measured via a water replacement method, in which the volume of the gas is equalised to the volume of the replaced water in the water pillar. The temperature and pressure were recorded to calculate the gas production at 35 ℃.

At the end of the co-substrate experiments, ca. 10 g of graphite granules with biofilm were sampled for scanning electron microscopy (SEM) imaging (JSM-IT500, Jeol, Japan). The granules were firstly rinsed with 0.1 mol L^**−**1^ phosphate buffer solution (PBS, pH 7.0, prepared with 0.058 mol L^**−**1^ Na_2_HPO_4_ and 0.042 mol L^−1^ KH_2_PO_4_, sterilised by 0.2 µm filtration) and then fixed in 2.5% glutaraldehyde (dissolved in PBS) for 17 h at 4 ℃. The treated samples were then rinsed with PBS and dehydrated with graded ethanol series solution (10%, 30%, 50%, 70%, 80%, 90%, 100%) for 10 min at 100 rpm agitation to fix the biofilm on the granules. The graphite granules were glued to an SEM specimen stub and carbon coated before imaging to increase the conductivity and avoid charging. Images were taken with a secondary electron detector at the acceleration voltage of 15 kV.

Carbon conversion efficiency was used to present the percentage of soluble end-products in the catholyte compared to the consumed substrates. Microbial growth as the biomass was not taken into consideration. The exact composition of the yeast extract is not available, and therefore yeast extract is not included in the carbon conversion efficiency calculation. Furthermore, the carbon originating from sodium 2-bromoethanesulfonate was less than 3% of the total carbon provided and, therefore, was not considered in the calculation of the carbon conversion efficiency. Carbon conversion efficiency was calculated by the following equation which followed the calculation from (Mohanakrishna et al. 2018):

1 $$Carbon\;conversion\;efficiency=\boldsymbol{ }\frac{{\sum }_{{\varvec{i}}}{{\varvec{n}}}_{({\varvec{i}},\boldsymbol{ }\boldsymbol{ }{\varvec{t}}+1)}-{\sum }_{{\varvec{i}}}{{\varvec{n}}}_{({\varvec{i}},\boldsymbol{ }\boldsymbol{ }{\varvec{t}})}}{{{\varvec{n}}}_{({\varvec{C}}{\varvec{D}},{\varvec{t}})}-{{\varvec{n}}}_{({\varvec{C}}{\varvec{D}},\boldsymbol{ }\boldsymbol{ }{\varvec{t}}+1)}+{{\varvec{n}}}_{({\varvec{M}}{\varvec{E}},\boldsymbol{ }{\varvec{t}})}-{{\varvec{n}}}_{({\varvec{M}}{\varvec{E}},{\varvec{t}}+1)}}\times 100$$

where *n*_*i*_ is the amount of product *i* at the sampling time *t* in moles, *n*_*CD*_ and *n*_*ME*_ are the amounts of the CO_2_ and methanol in moles after substrate addition at the sample time *t*, respectively, and *t* + 1 represents the following sampling time before the substrate addition. Two and four moles of carbons are obtained from one mole of acetate and butyrate, respectively. As CO_2_ could be left unused in both catholyte and/or in the gas phase, *n*(CD, _*t*+1_) was subtracted as the lost carbon. The potential losses of VFAs were calculated by comparing the VFA concentrations from before and after the substrate(s) additions as well as the removed VFAs due to the sampling. Therefore, concentrations of the VFAs are reported in the main text, while amounts of VFAs in mmol are used for the carbon conversion efficiency and electron efficiency calculations. The amount of VFAs in mmol is separately reported in the supplementary information. The results in the text are given as the average value from duplicated reactors and with standard deviations.

Electron efficiency (EE) was calculated with Eq. 2, where *f*_*e*,*i*_ represent the molar conversion factor of product *i*. For the products, 8 electron equivalents were required for the formation of one mole of acetate, 20 electron equivalents for the formation of one mole of butyrate, and 2 electron equivalents for the formation of one mole of hydrogen gas. For the electron sources, the electrons from the electrode are derived by integrating the recorded current over time. The electrons from methanol are calculated as 6 electron equivalents per mole of methanol. *F* is Faraday constant (96,485 C mol^−1^ electron), and *Ι* is current (A).

2 $$EE=\frac{{\varvec{F}}\boldsymbol{ }\times \left({\sum }_{{\varvec{i}}}{{\varvec{n}}}_{({\varvec{i}},{\varvec{t}}+1)}-{\sum }_{{\varvec{i}}}{{\varvec{n}}}_{({\varvec{i}},{\varvec{t}})}\right)\times {{\varvec{f}}}_{{\varvec{e}},{\varvec{i}}}}{\int {\varvec{I}}\boldsymbol{ }{\varvec{d}}{\varvec{t}}+{\varvec{F}}\times \left({{\varvec{n}}}_{({\varvec{M}}{\varvec{E}},{\varvec{t}})}-{{\varvec{n}}}_{({\varvec{M}}{\varvec{E}},{\varvec{t}}+1)}\right)\times {{\varvec{f}}}_{{\varvec{e}},{\varvec{M}}{\varvec{E}}}}\times 100$$

### Characterisation of the microbial culture

Samples of planktonic cells for the microbial community analysis were collected at the end of each batch of the enrichment and at the end of the co-substrate experiment. At the end of each batch, 40 mL of the cathodic broth was collected and centrifuged (4000 rpm, 20 min), and the resulting pellets were resuspended into 1 mL PBS (0.1 mol L^−1^) and stored at − 80 ℃. For the biofilm samples, ca. 15 g of graphite granules at the end of the co-substrate experiment were collected and gently washed three times with PBS to remove planktonic cells. The graphite granules were then soaked in 40 mL PBS and ultrasonicated for 3 min (USC 300 T, VWR, USA). The supernatant was collected, and the ultrasonication of the granules was repeated with fresh PBS. After three repetitions, all the collected supernatant was centrifuged, and the pellet was stored in 1 mL PBS at − 80 °C. DNA was extracted by using the DNeasy kit PowerSoil Pro Kit (Qiagen, Germany) as per the manufacturer’s instructions and quantified using a NanoDrop spectrophotometer (NanoDrop™ 1000, Thermo Fisher Scientific, USA).

The eubacterial 16S rRNA gene copies were quantified by quantitative polymerase chain reaction (qPCR) analysis of the cathodic biofilm and planktonic samples from the end of the co-substrate experiment. The amplification was conducted by Bio-Rad CFX96 (CFX96 Touch Real-Time PCR Detection System, Bio-Rad, USA). The qPCR conditions were 95 ℃ for 10 min, 34 cycles of 95 ℃ for 15 s, and 62 ℃ for 60 s. Primer pairs 338F/518R were used targeting the V3-V4 region of eubacterial 16S rRNA gene (Fierer et al. 2005). SYBR Green master mix (Maxima SYBR Green/ROX qPCR Master Mix, Thermo Fisher Scientific, USA) was used for all qPCR reactions. A volume of 25 µL was prepared for the amplification, containing 0.5 µM of each primer, diluted sample DNA, and RNase-free sterile water. A tenfold dilution series of the plasmid DNA standard (Rinta-Kanto et al. 2016) was used to plot a standard curve with concentrations ranging from 3 × 10^6^ to 3000 copies per reaction to the threshold cycle (Ct).

Both the planktonic and biofilm samples were sequenced by the Ilumina Novaseq 6000 platform at a depth of 100 K (Novogene, UK). The V3-V4 region of the 16S rRNA genes was PCR amplified with the primer pair 341F/806R (5’-3’ sequence: CCTAYGGGRBGCASCAG/GGACTACNNGGGTATCTAAT). The sequenced results were processed using the DADA2 pipeline with the qiime2 software 2021.4 (Bolyen et al. 2019). The taxonomic analysis was conducted by referring to the pre-trained classifier Greengenes 13_8 99% OTUs full-length sequences (Bokulich et al. 2018).

The data obtained during this study was deposited at the European Nucleotide Archive under project accession number PRJEB61945. The abundant OTUs were used for Megablast to search within the NCBI nucleotide database to provide extended interpretation beyond the genus level. Subsequently, beta diversity metrics (weighted-Unifrac dissimilarity) and principal coordinate analysis (PCoA) were calculated using the q2-diversity plugin. Microbial communities were statistically compared using permutational analysis of variance (PERMANOVA) (Anderson 2008).

## Results

### Enrichment of microbial culture utilising methanol and CO_2_

**Fig. 1:**
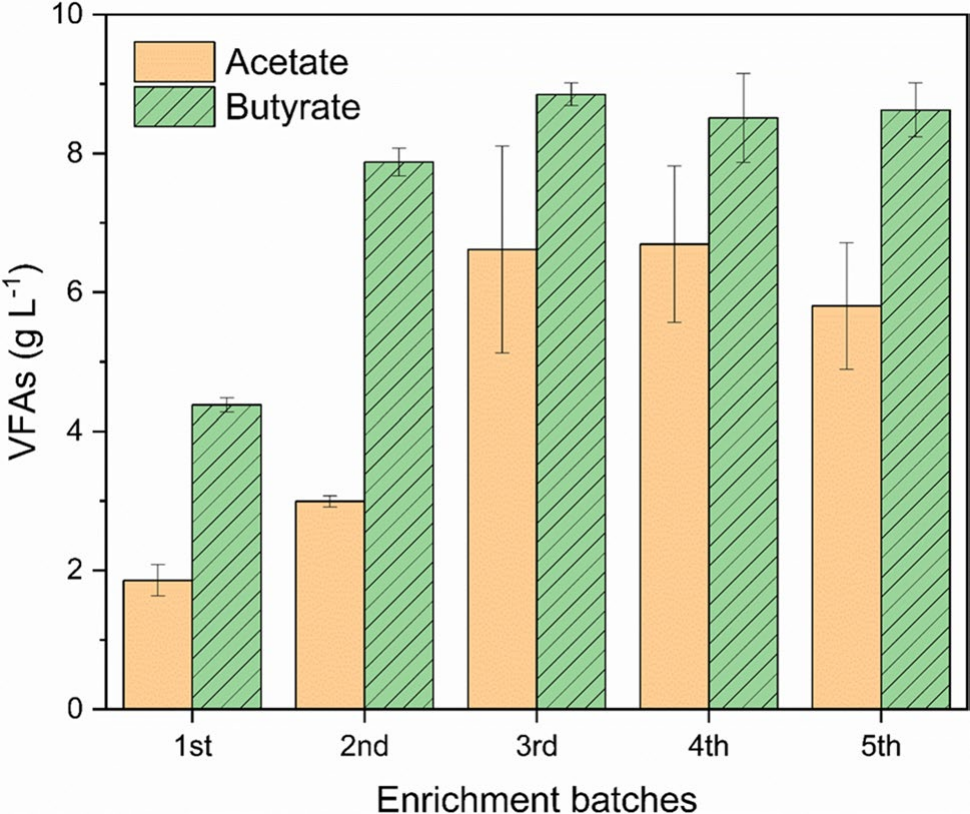
Production of VFAs at the end of each enrichment batch. Error bars indicate the standard deviations in duplicate reactors

A mixed microbial culture was selectively enriched for butyrate production from methanol and CO_2_ in MES (Fig. 1, Fig. S2). The enrichment started with solely CO_2_ feeding for 4 days (30 min feeding for two times), which resulted in an immediate VFA production reaching acetate and butyrate concentrations (in this study, only n-butyrate was detected, hereinafter referred to as butyrate) of 3.5 ± 0.6 g L^−1^ and 0.36 ± 0.05 g L^−1^, respectively (Fig. S3). From day 5 onwards, methanol was periodically added together with CO_2_. Acetate was stabilised at a concentration of ca. 1.8 g L^−1^ throughout the first batch, while butyrate concentration continuously increased and reached the maximum titre of 5.8 ± 0.1 g L^−1^ on day 22. Afterwards, a decrease in the butyrate concentration was observed, and 4.4 ± 0.1 g L^−1^ of butyrate was obtained at the end of the first batch (Fig. S3). Similar VFA production pattern was observed during the following four batches. Namely, acetate was first produced until the concentration reached ca. 1.8 g L^−1^, after which a sequential butyrate production was initiated. A maximum butyrate titre observed in the second batch was 8.0 ± 0.2 g L^−1^, and further increases in the maximum butyrate titres were achieved in the batches three to five, varying between 9.8 ± 0.4 and 10.6 ± 0.1 g L^−1^. The average butyrate production rate in the last three batches was 0.17 ± 0.05 g L^−1^ d^−1^. In addition to acetate and butyrate, a longer chain VFA, caproate, was also observed in all enrichment batches with the maximum titres ranging between 0.22 and 0.50 g L^−1^ (Table S3).

### MES performance with different substrate feedings

#### VFA production

**Fig. 2:**
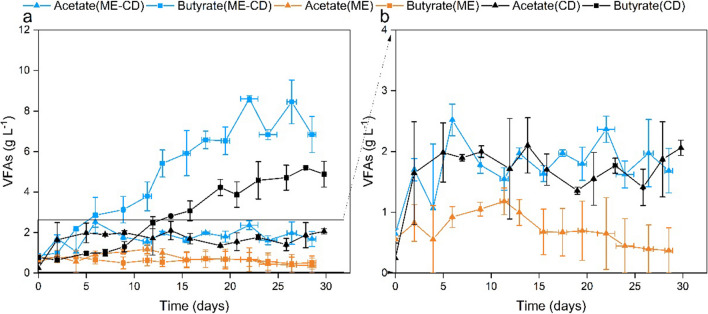
Concentrations of VFAs in the co-substrate addition experiments with **a** the overview of all detected VFAs (acetate and butyrate) and **b** the acetate production. ME-CD (fed with methanol and CO_2_); ME (fed with only methanol); CD (fed with only CO_2_). Error bars are the standard deviations in duplicate reactors

Acetate and butyrate were the observed VFAs in the co-substrate addition experiments (Fig. 2a, Fig. S4). MES fed with methanol and CO_2_ (ME-CD) resulted in a significant enhancement in butyrate production (ANOVA, *p* < 0.05, Table S4), outperforming the MES fed with methanol (ME) and CO_2_ (CD). In ME, a slight increase of acetate concentrations was observed from day 0 to day 9, with the concentration increasing from 0.56 ± 0.02 to 1.05 ± 0.11 g L^−1^. However, the butyrate concentration dropped from 0.77 ± 0.15 g L^−1^ (day 0) to 0.50 ± 0.29 g L^−1^ (day 9). No VFA production was obeserved after day 12 although methanol was consumed (Fig. 4b). Methanol, as the sole substrate, was not utilised for the VFA production in MES, and thus, the results are not further included in the following discussion. When comparing ME-CD to CD, similar acetate production pattern was observed in both groups, where acetate production initiated immediately from the beginning of the run and reached the threshold concentration (ca. 1.8 g L^−1^) on day 2 (Fig. 2b). Acetate concentration then fluctuated between 1.7 and 2.0 g L^−1^ throughout the operation. The main difference between ME-CD and CD was in butyrate production. In CD, butyrate production initiated from day 2 and reached the maximum butyrate titre (5.2 ± 0.1 g L^−1^) on day 28 with the production rate of 0.20 ± 0.03 g L^−1^ d^−1^. From day 2 onwards, VFA production in ME-CD was dominated by butyrate with a notable production rate of 0.36 ± 0.01 g L^−1^ d^−1^ until the maximum butyrate titre of 8.6 ± 0.2 g L^−1^ was reached on day 22. A decrease in the VFA concentration in ME-CD was observed after day 22 due to the CO_2_ sparging (the concentration of VFAs decreased after sparging), which resulted in the final acetate and butyrate titres of 1.7 ± 0.4 g L^−1^ and 6.9 ± 0.9 g L^−1^, respectively. However, no notable loss of VFAs was observed in CD. Overall, the addition of methanol as a co-substrate with CO_2_ boosted the maximum butyrate titre by 1.7-fold and the butyrate production rate by 1.8-fold.

#### H_2_ production and pH

**Fig. 3:**
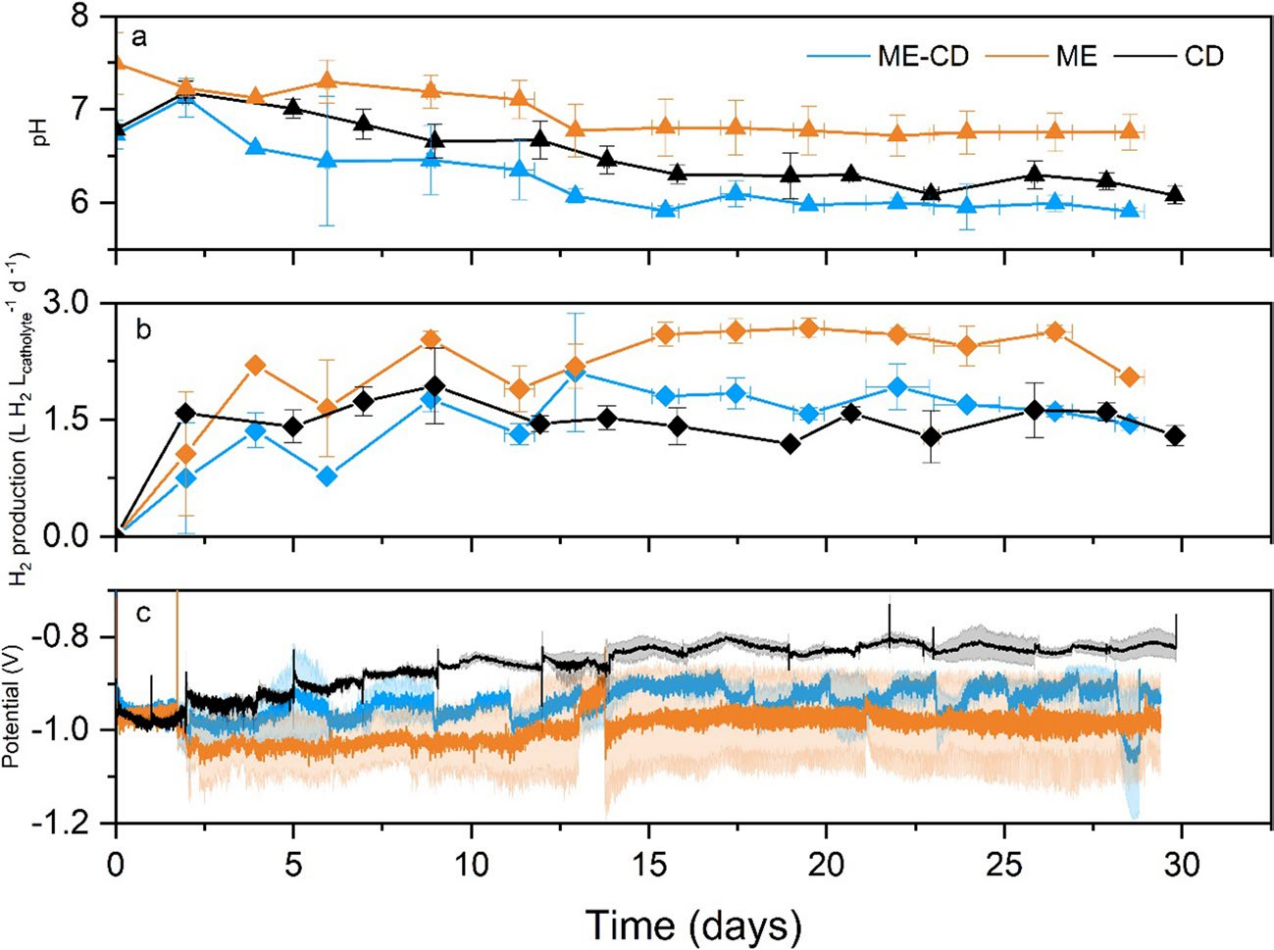
Cathodic pH (a), H_2_ production rates (b), and cathodic potential (c) during the co-substrate addition experiments. ME-CD (fed with methanol and CO_2_); ME (fed with only methanol); CD (fed with only CO_2_). Error bars indicate the standard deviations in duplicate reactors

The role of methanol in MES was further revealed through the co-substrate addition experiment. Gradual decrease in pH was observed in both ME-CD and CD (Fig. 3a) due to the CO_2_ sparging and accumulation of VFAs, from which ME-CD resulted in the lowest pH values during the operation reaching value of 6.0 on day 14, after which it was manually maintained between 6.0 and 6.2 until the end of the operation. In CD, the pH also gradually decreased and ranged from 6.2 to 6.5, while in ME, no notable decrease of pH was observed (Fig. 3a). The cathodic potentials showed different profiles among the three experimental groups with the set current of − 100 mA (Fig. 3c). In the beginning of the experiments, similar cathodic potential (ca. − 0.95 V) was recorded. The cathodic potential of CD gradually increased to between − 0.8 and − 0.85 V. However, lower cathodic potentials were recorded in ME-CD (between − 0.90 and − 1.0 V) and ME (between − 1.0 and − 1.1 V). H_2_ production was a crucial step in MES, which assisted the CO_2_ reduction. During the operation, H_2_ was continuously produced and accumulated in the cathodic headspace as well as the gas bags (Fig. 3b). CD resulted in the lowest H_2_ accumulation with an average rate of 1.5 ± 0.1 L H_2_ L_catholyte_^−1^ d^−1^, while H_2_ accumulated in ME-CD and ME at higher rates, 1.6 ± 0.1 L H_2_ L_catholyte_^−1^ d^−1^ and 2.1 ± 0.3 L H_2_ L_catholyte_^−1^ d^−1^, respectively.

#### Carbon and electron conversion efficiencies

**Fig. 4:**
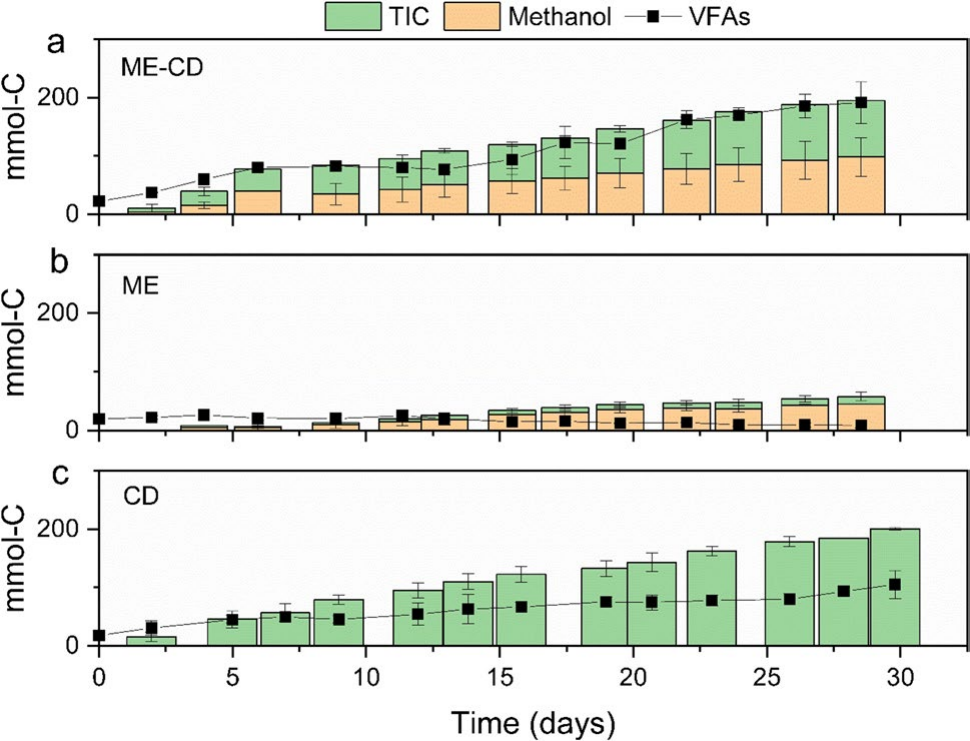
Substrate consumption and VFA production with different carbon sources. Figures a, b, and c represent the reactors fed with CO_2_ + methanol (ME-CD), methanol (ME), and CO_2_ (CD), respectively. Error bars indicate the standard deviations in duplicate reactors

The total carbon that was consumed as the main carbon sources, i.e. methanol and CO_2_ (CO_2_ is referred to as total inorganic carbon (TIC) in this section), was compared to the total carbon present in the produced VFAs (Fig. 4). In CD, altogether 200.7 ± 2.1 mmol-C TIC (equivalent to 2.4 ± 0.03 g-C) was utilised with the rate of 0.08 ± 0.002 g-C d^−1^, of which 39.4 ± 9.2% of the carbon was recovered in VFAs. In ME-CD, a total of 99.4 ± 24.1 mmol-C TIC (equivalent to 1.2 ± 0.3 g-C) as CO_2_ was utilised with the average rate of 0.05 ± 0.01 g-C d^−1^, while methanol was also utilised in the similar rate (ca. 0.05 g-C d^−1^) and resulted in the overall consumption of methanol as 98.3 ± 32.9 mmol-C (equivalent to 1.2 ± 0.4 g-C). After compensating the losses of VFAs due to substrate addition and sampling, 101.8 ± 4.9% of the carbon sources (TIC + methanol) in ME-CD were recovered in VFAs, of which 86.3 ± 4.4% was recovered in butyrate. Although no accumulation of methanol and TIC was obtained at the end of the operation, methanol was observed to remain available between some of the two consecutive feeding timepoints (e.g. days 5 to 7 in ME-CD1, Table S5 and S6) while TIC was always completely depleted.

In ME, methanol was consumed at a rate of 0.018 ± 0.0009 g-C d^−1^, which resulted in a 42.5 ± 2.3 mmol-C consumption until day 29 (Fig. 4b). As smaller methanol consumption was observed in the abiotic experiment (0.005 g-C d^−1^ during 7 days, Table S7), the methanol consumption in ME was likely related to the microbial activity, as the biofilm growth was observed by qPCR **(**Fig. S5). In addition, trace amounts of TIC were observed in ME, with an overall consumption of 12.7 ± 7.4 mmol-C (equivalent to 0.15 ± 0.09 g-C) (Table S5). Although methanol was the sole added carbon source in ME, the TIC present in the catholyte could originate from the oxidation of the VFAs (originating from the inoculum) or methanol oxidation to support the microbial growth in ME (Fig. S5).

**Fig. 5:**
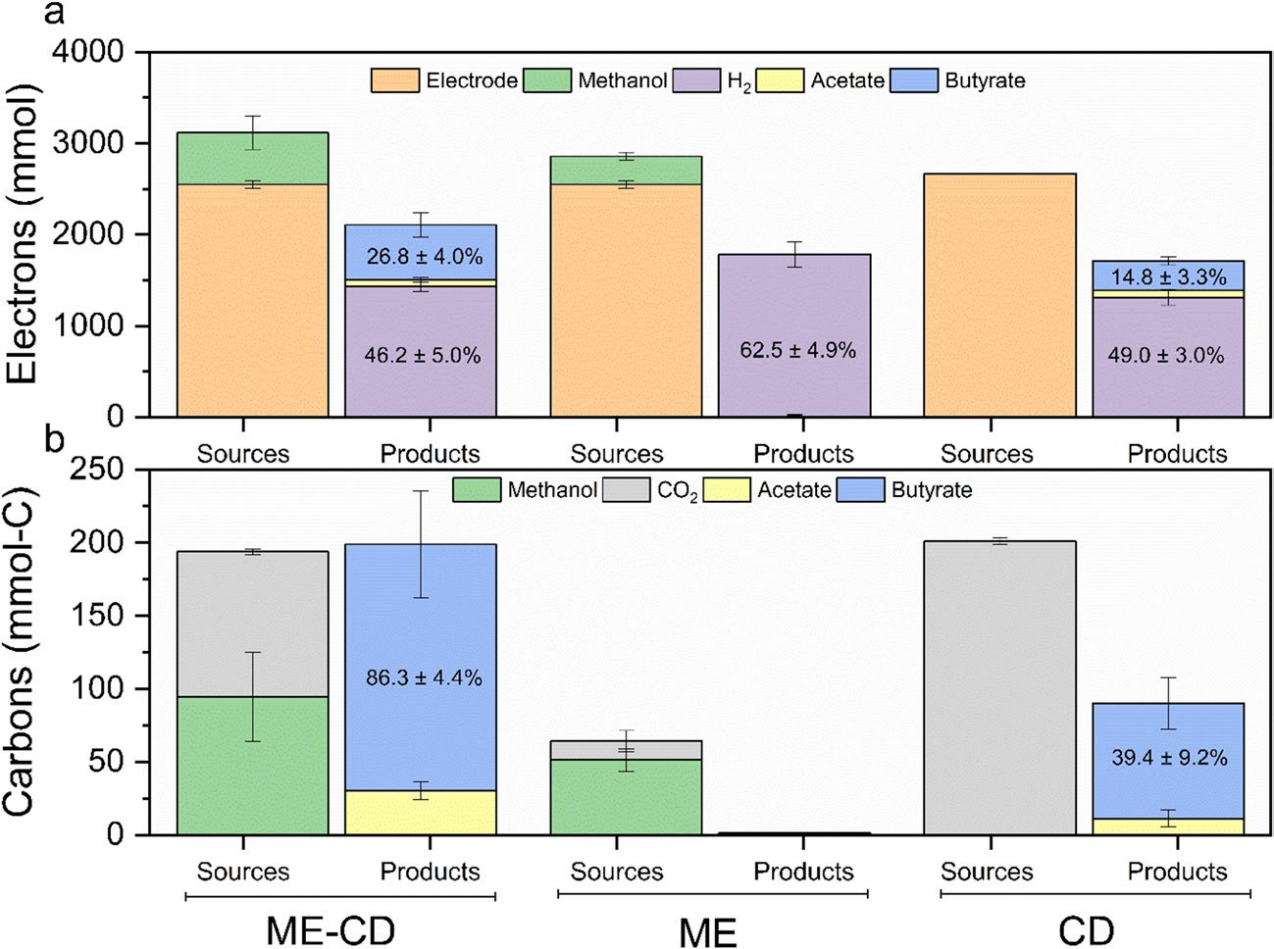
Electrons (**a**) and carbons (**b**) present in the inputs and in the products. Green blocks represent the electrons/carbon provided by overall consumption of methanol, grey blocks the carbon provided by consumed CO_2_, and orange blocks the electrons provided by electrode. ME-CD (fed with methanol and CO_2_); ME (fed with only methanol); CD (fed with only CO_2_). The percentages of carbon or electron recovery in H_2_ or butyrate are shown the figures. The electron recoveries (a) in acetate were 3.8 ± 0.5% (ME-CD) and 1.7 ± 0.9% (CD). The carbon recoveries (b) in acetate were 15.5 ± 0.5% (ME-CD) and 5.5 ± 2.8% (CD). Error bars indicate the standard deviations in duplicate reactors

The distributions of electrons and carbon in the electron sources (methanol and electrode) and in the products (acetate, butyrate, and H_2_) were plotted to have an insight on the electron flow and the role of methanol, respectively, in the co-substrate addition experiment (Fig. 5). Thus, the overall efficiency of the process can be assessed. The overall electron efficiency for ME, CD, and ME-CD were 61.5 ± 6.4%, 65.4 ± 5.4%, and 76.9 ± 0.6%, respectively. The majority of the electrons were recovered in H_2_ in all three experimental groups. Overall, 62.5 ± 4.9%, 49.0 ± 3.0%, and 46.2 ± 5.0% of the electrons were recovered in H_2_ in ME, CD, and ME-CD, respectively. Butyrate production contributed to 14.8 ± 3.3% and 26.8 ± 4.0% of the electron recovery in CD and ME-CD, respectively.

### Cathodic microbial cultures

Planktonic cells and biofilms were analysed to gain knowledge of the microbial communities in MES. The microbial culture during the enrichment was adapted in five batches. *Eubacterium* was always the most dominant genus and its relative abundance increased from 42.2 ± 1.0% (first batch) to 55.0 ± 4.5% (fifth batch), while for the second most enriched genus, i.e. *Bacteroides*, the relative abundance decreased from 26.5 ± 1.5% (first batch) to 12.6 ± 4.0% (fifth batch).

**Fig. 6:**
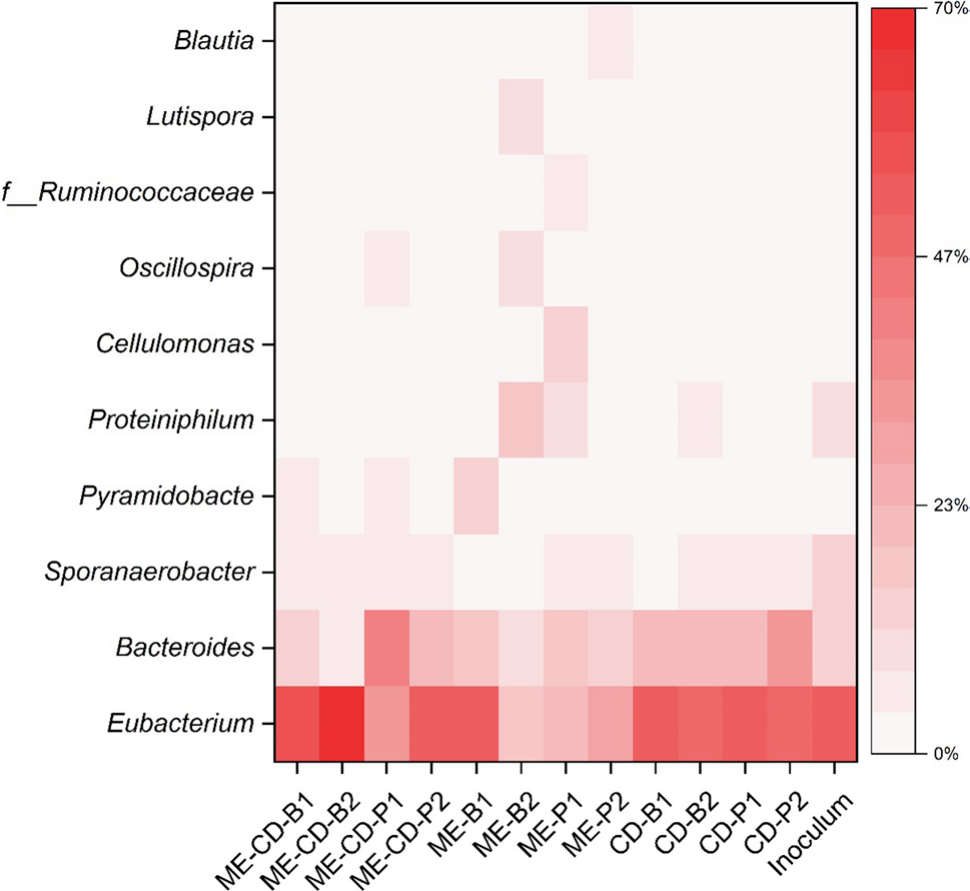
Heat map and the relative abundance of the ten most dominant genera fed with CO_2_ (CD), methanol (ME), and CO_2_ + methanol (ME-CD) in the co-substrate experiment (B, biofilms; P, planktonic cells, 1 and 2 represent the microbial culture from each duplicate). *f__Ruminococceae* is an unknown genus belonging in the family *Ruminococceae.* Inoculum represent the average of five enrichment batches (samples were taken from the planktonic cells at the end of each enrichment culture, error bars are not shown in the heatmap)

In the co-substrate experiments, higher microbial growth was observed in CD compared to ME-CD and ME (Fig. S5). A total of 942 OTUs were observed. Relative abundances of the ten most abundant genera are shown in the heatmap (Fig. 6). The most enriched genus was *Eubacterium*. *Eubacterium* was predominantly enriched in the VFA-producing cultures with the relative abundance of 50.6 ± 2.5% (CD) and 52.6 ± 2.5% (ME-CD) and was less present in the ME with the relative abundance of 30.5 ± 13.0%. In CD, the relative abundance of *Eubacterium* between biofilm and planktonic cells remained similar (50.4 ± 1.4% and 50.8 ± 3.3%, respectively). However, in the methanol-fed cultures (ME and ME-CD), *Eubacterium* was more enriched in the biofilm (ME-CD: 63.5 ± 6.7% and ME: 35.5 ± 16.4%) than in the planktonic cells (ME-CD: 42.1 ± 9.6% and ME: 25.5 ± 4.1%), respectively. Generally, smaller variations in the parallel microbial culture compositions were observed in the biofilm cells in ME-CD and CD. The presence of the biofilm on the graphite granules from all experimental groups was confirmed with SEM (Fig. S6). Genus *Bacteroides* was also present in all experimental groups with a relative abundance of 20.5 ± 4.4% (ME-CD), 24.1 ± 4.4% (CD), and 14.2 ± 2.9% (ME). Unlike *Eubacterium*, the relative abundance of *Bacteroides* between biofilm and planktonic cells remained similar in ME, but showed differences in the CO_2_-fed cultures (ME-CD and CD) where *Bacteroides* was more enriched in the planktonic cells (ME-CD: 30.6 ± 9.4% and CD: 27.2 ± 4.4%) than the biofilm (ME-CD: 10.5 ± 3.9% and CD: 21.1 ± 1.1%).

There was no statistical difference in terms of the richness and evenness among the three experimental groups. However, microbial communities that were plotted in PCoA indicated that ME was distinct from CD and ME-CD (Fig. S7) and the difference was further validated by the PERMANOVA (*p* < 0.05). ME-CD and CD were identified to have similar microbial communities, in which *Eubacterium* and *Bacteroides* are well-known microorganisms for VFA production. The most dominant OTU was further queried by the NCBI megablast in the 16S rRNA sequences database, and the results revealed a high similarity to *Eubacterium limosum* (100% similarity, 100% query cover) and *Eubacterium callanderi* (100% similarity, 100% query cover) (Table S8).

## Discussion

### Role of methanol as a co-substrate in MES

Adding methanol enhanced the butyrate production in MES, increasing the butyrate production rate by 1.7-fold (production rate of 0.3 ± 0.01 g L^−1^d^−1^) and maximum titres by 1.8-fold (8.6 ± 0.2 g L^−1^), while the acetate titres remained similar compared with only CO_2_ feeding. The overall carbon conversion efficiency for the VFA production was also enhanced to 101.8 ± 4.9% with methanol addition (compared to 39.4 ± 9.2% with CO_2_ feeding) of which methanol and CO_2_ contributed equally, ca. 50% of carbon (Fig. 5b**)**. The high carbon conversion efficiency (over 100%) could be explained by the following aspects: The amount of carbon from yeast extract cannot be quantified due to its unknown chemical composition which could serve as extra carbon source. Moreover, the migration of methanol and VFAs to the anode cannot be quantified accurately as the anode was an open system, and the evaporation of methanol and VFAs could introduce experimental errors.

For those acetogens which can utilise methanol as the carbon and energy source, it is commonly recognised that part of the methanol is utilised to reverse the WLP to provide reducing equivalents for the reduction of CO_2_ (Kremp and Müller 2021). The overall stoichiometric equation for methanol utilisation is shown in Eq. 3. First, every one out of four moles of methanol is completely oxidised to CO_2_ to provide six moles of electrons, which are utilised for the reduction of three moles of CO_2_ to CO. The three moles of CO are coupled with the three moles of remaining methanol and produce acetyl-CoA in WLP (Kremp and Müller 2021), which can be converted to acetate through phosphorylation resulting in ATP generation (May et al. 2016). For *E. limosum,* butyrate production has also been reported from methanol and CO_2_. Another mole of methanol needs to be oxidised to provide extra reducing equivalents to convert three moles of acetyl-CoA to butyryl-CoA via the reverse β-oxidation. The CoA of butyryl-CoA is transferred to an acetate via the butyryl-CoA:acetate CoA transferase for the synthesis of butyrate (Kremp and Müller 2021) with reaction shown in Eq. 4.

3 $$4 C{H}_{3}OH + 2 C{O}_{2}\to 3 C{H}_{3}COOH + 2 {H}_{2}O$$

4 $$5 C{H}_{3}OH + C{O}_{2}\to 1.5 C{H}_{3}C{H}_{2}C{H}_{2}COOH + 4 {H}_{2}O$$

Methanol has been reported to improve the H_2_/CO_2_ utilisation in *Eubacterium limosum*, resulting in a higher growth rate and H_2_ consumption rate (Kim et al. 2021). In addition, the energy efficiency for the bioproduction of acetate from methanol under anaerobic conditions is higher compared to the gaseous substrates, e.g. H_2_ (Claassens et al. 2019). When utilising methanol as a substrate, more electrons are directed to products for the generation of sufficient cellular energy in the form of ATP (Claassens et al. 2019) and result in higher product yields, which is in accordance with the higher carbon efficiency achieved in ME-CD than CD in this study.

Methanol metabolism in MES could involve both methanol oxidation (releasing six electrons) and/or transfer of the electrons to acetyl-CoA without extracellular electron transfer as discussed above. In order to investigate whether methanol was used as the electron donor in ME-CD, the electron recovery was first calculated considering electrode as the sole electron source. The electron recoveries in butyrate, acetate, and H_2_ were 93.8 ± 4.6%, 65.4 ± 5.4%, and 68.8 ± 5.9% for ME-CD, CD, and ME, respectively. Specifically for ME-CD, at a maximum 133.1 ± 1.0% electron recovery was observed between two consecutive substrate-feeding timepoints (days 15 to 17), which implied the potential role of methanol as another electron source in ME-CD (Fig. S8). If the electrons provided by methanol are also considered, the electron recoveries decrease to 76.9 ± 0.6% in ME-CD and 61.5 ± 6.4% in ME. Methanol oxidation in acetogens can be a series of reversed WLP reactions (from methanol to CO_2_) (Kremp and Müller 2021). In this study, CO_2_ and reducing equivalents (e.g. H_2_) were both supplied. Thus, the methanol oxidation in MES was likely regulated by the availability of the reducing equivalents (such as the H_2_ partial pressure) and CO_2_ (Pacaud et al. 1985), as well as the characteristics of the microorganism (such as the intracellular NADH/NAD^+^ pool) (Jeong et al. 2015). In this research, the role of methanol can be concluded as both the carbon and electron donor. However, the metabolism of methanol in MES could be affected by several factors and needs to be further investigated.

### Butyrate production in MES

Butyrate is a value-added product (market price of ca. 1500 USD/ton), with diverse applications, for example, in food and pharmaceutical industries. The current butyrate synthesis is mainly via chemical synthesis which requires the use of crude oil. In addition, butyrate can be synthesised via fermentation from sugars (Dwidar et al. 2012). In this work, the production of butyrate from methanol and CO_2_ is considered beneficial as methanol is a bulk chemical with the market price of ca. 300 USD/ton and it can be synthesised from renewable sources or utilised from industrial side streams.

Butyrate production in MES is often accompanied with the consumption of acetate. In this study, an acetate threshold concentration (ca. 1.8 g L^−1^) was necessary to promote butyrate production, which has also been reported with a slightly higher acetate threshold concentration (2.0 to 4.0 g L^−1^) (Jourdin et al. 2019). The acetate threshold concentration was observed in both ME-CD and CD and is likely determined by the enriched microbial communities (*E. limosum* dominating mixed culture), as the certain amount of acetate is suggested to be needed for the butyrate production with *E. limosum* (Kremp and Müller 2021)*.*

The first reported butyrate production in MES was rather low, with the maximum butyrate concentration of 0.44 g L^−1^ and a production rate of 0.04 g L^−1^ d^−1^ (Ganigué et al. 2015). The maximum butyrate titre and production rate were further increased to 1.9 g L^−1^ and 0.16 g L^−1^ d^−1^, respectively, by adjusting the feeding strategy of CO_2_ to maintain a high H_2_ partial pressure (Batlle-Vilanova et al. 2017). In addition, through the continuous feeding of CO_2_, butyrate production was reinforced and resulted in the butyrate production rate of 3.2 ± 0.1 g L^−1^ d^−1^ and butyrate titre of 9.3 g L^−1^ (Jourdin et al. 2019). In addition to CO_2_ as the sole carbon source, butyrate production in MES has been enhanced by adding other electron donors, such as formate, ethanol, or lactate (Izadi et al. 2021b; Li et al. 2022; Zhang et al. 2023). Formate addition increased the maximum butyrate titre in MES by 3.8-fold (to 0.14 ± 0.02 g L^−1^), while ethanol addition increased the product diversity (i.e. the production of butanol and hexanol) but not the butyrate production rates and titres (Izadi et al. 2021b). Concurrent addition of lactate and ethanol (5 g L^−1^ each) resulted in a high butyrate production rate (0.9 g L^−1^ d^−1^) and a maximum butyrate titre of 6.3 g L^−1^ (Zhang et al. 2023). In this study, methanol addition resulted in a selective production of butyrate in MES with the maximum titre of 8.6 ± 0.2 g L^−1^ (one of the highest values reported in MES), and production rate of 0.36 ± 0.01 g L^−1^d^−1^, which likely remained rather low due to the insufficient carbon source (further discussed in the next section).

### Limiting factors of MES fed with methanol

Although both CO_2_ (TIC) and methanol in the liquid phase were completely consumed by the end of the experiments in ME-CD (total consumption of both substrates was ca. 99 mmol-C), there was occasionally accumulation of methanol between the substrate feedings. In this study, CO_2_ was fed in semi-batch mode (ca. 6 L CO_2_ was purged through the medium each time). Most of the CO_2_ was sparged through the system, and only ca. 5% of the CO_2_ remained in the liquid phase as the carbon source (Table S5). TIC, as the main electron acceptor in catholyte, was completely consumed between each substrate feeding, while the electron donors (methanol and H_2_) remained available indicating that CO_2_ was the limiting substrate in ME-CD, CD, and ME. Continuous feeding of CO_2_ would provide sufficient carbon source/electron acceptor in MES, and it has been reported as an efficient strategy to increase the production rate of both acetate and the longer chain fatty acids, such as butyrate and caproate, in MES (Batlle-Vilanova et al. 2016; Jourdin et al. 2019; Izadi et al. 2021a). In this study, however, the optimisation of CO_2_ utilisation was not the main aim and should be improved in future studies.

The VFA production was also affected by the pH, which, in this study, had the most direct influence on the dissolution of TIC. The lower pH in ME-CD (pH 6.0 to 6.2) than in CD (pH 6.2 to 6.4) resulted in lower TIC values in ME-CD after CO_2_ sparging (ca. 4.3 mM-C in ME-CD vs. ca. 7.5 mM-C in CD) and subsequently limited the VFA production from CO_2_ in ME-CD as discussed earlier. The slightly acidic pH (5.8) has been reported to increase the acetate production rate with a mixed microbial culture (Batlle-Vilanova et al. 2016). However, the optimal pH for the growth of *E. limosum* is close to neutral, and thus, slightly acidic conditions may limit the growth of *E. limosum* (Pacaud et al. 1985), which could explain the lower microbial growth in ME-CD compared to CD (Fig. S5). On the other hand, microbial chain elongation is also affected by pH. For example, in methanol-based chain elongation, the reactor pH facilitated the changes in product selectivity, where pH 6.75 resulted in n-butyrate production while i-butyrate production was favoured at pH 5.25 (De Leeuw et al. 2020). In this study, the pH was maintained at above 6 to avoid the potential toxicity due to the VFA accumulation, while under a more acidic pH, the product spectrum could likely be expanded (e.g. i-butyrate production). However, if the pH is elevated, the dissolution of TIC will be higher which also affects the VFA production (Pacaud et al. 1985). Thus, both the CO_2_ feeding and pH should be optimised to enhance the production rates and titres and to alter the product spectrum in MES fed with methanol.

### Microbial culture for butyrate production in MES

Butyrate was the main product in both ME-CD and CD, which is attributed to the microbiome with *Eubacterium* as the dominant genus. Especially, the enrichment of *Eubacterium* in the biofilm samples (relative abundance in biofilm was 63.5 ± 6.7% in ME-CD and 50.4 ± 1.4% in CD) suggests that the biofilm played a more important role than planktonic cells in the production of VFAs. *E. limosum* and *E. callanderi* are the two species that shared the highest similarity compared to the most abundant OTUs in this study. *E. limosum* is a well-known acetogen that grows on methanol and/or CO_2_ with acetate and butyrate as the main metabolites (Pacaud et al. 1986). *E. callanderi* was originally reported unable to utilise one-carbon compounds (e.g. methanol and CO_2_) (Mountfort et al. 1988). However, the *E. callanderi* KIST612 strain, formerly known as *E. limosum* KIST612, is reported to utilise methanol as well as other C1 compounds (Dietrich et al. 2021).

The inoculum for the experiments was cultivated with methanol and CO_2_ over a period of one year before the co-substrate experiments. When comparing the inoculum used in the co-substrate experiments after the enrichment period to the original culture fed with only CO_2_, the relative abundance of *E. limosum* increased from 11.2% (Vassilev et al. 2024) to 52.2%, likely due to the methanol addition. In this study, feeding with only CO_2_ resulted in similar microbial culture with the similar *E. limosum* relative abundances than reactors fed with both CO_2_ and methanol, which is likely due to the fact that the inoculum was fed with methanol and CO_2_ for a long time period. However, when methanol accumulated in the cathode in the reactors fed with methanol only, the high concentration of methanol likely hindered the growth of *E. limosum* as the relative abundances decreased compared to ME-CD and CD reactors.

For *E. limosum*, it is reported that the butyrate production is dependent on the carbon and energy sources (Litty and Müller 2021). Feeding with H_2_ and CO_2_ only in batch bottles resulted in the production of acetate, while butyrate production was achieved with methanol and CO_2_ feeding. However, acetate has usually been the main product with acetate: butyrate ratio of 1:0.33 growing with C1 feedstocks and their mixtures reported with *E. limosum* (Litty and Müller 2021). In this study, the product spectrum was dominated by butyrate, which could be due to the dual role of the supplied current, which not only provides electrons for the bacterial metabolism, but may also affect the extracellular oxidation–reduction potential and thus, may alter the product spectrum (Liu et al. 2013). Short batch bottle experiments done in this study (described in supplementary information and fed with methanol and CO_2_, without electricity) indicated that acetate would be the main product, reaching 1.7 ± 0.02 g L^−1^ on day 6 accompanied with butyrate production (0.59 ± 0.01 g L^−1^) **(**Fig. S9**)**. From days 6 to 8, methanol addition led to the microbial growth (OD_600_ increased from 0.62 ± 0.08 to 0.97 ± 0.05), while acetate concentration remained at 1.7 ± 0.3 g L^−1^ and butyrate concentration slightly increased to 0.66 ± 0.10 g L^−1^.

*Eubacterium* dominating cultures are widely reported in methanol-based chain elongation reactors, where it is suggested to be the responsible genus for butyrate production from acetate and methanol (Chen et al. 2016, 2020; De Leeuw et al. 2020). However, in MES *Eubacterium*-dominating cultures are rarely reported (Blasco-Gómez et al. 2019; Zhao et al. 2023). This study showed for the first time the potential of *Eubacterium* as the core microorganism in MES, which would result in the production of mainly butyrate from only CO_2_ or a mixture of CO_2_ and methanol.

In conclusion, methanol was employed for the first time as a co-substrate with CO_2_ in MES resulting in an increase of butyrate production rates by 1.7-fold (up to 0.36 ± 0.01 g L^−1^ d^−1^) and titres by 1.8-fold (up to 8.6 ± 0.2 g L^−1^) compared to CO_2_ as the sole substrate. Methanol acted both as carbon and electron source for VFA production. *Eubacterium* was the dominant genus, known to produce butyrate from CO_2_ as well as from CO_2_ and methanol. Overall, this study introduces a promising strategy to enhance the bioproduction of butyrate.

## Supplementary Information

Below is the link to the electronic supplementary material.

Supplementary file1 (PDF 1425 KB)

## Data Availability

The datasets generated during and/or analysed during the current study are available from the corresponding author on reasonable request. The sequence data generated during the current study is available in the European Nucleotide Archive under project accession number PRJEB61945.
