# Supplementary material for: Methanol as a co-substrate with CO2 enhances butyrate production in microbial electrosynthesis
Source: Appl Microbiol Biotechnol. 2024 Jun 14;108:372. doi: 10.1007/s00253-024-13218-y (PMC11178620; doi:10.1007/s00253-024-13218-y)
Supplement: Supplementary file 1 [file 253_2024_13218_MOESM1_ESM.pdf]

Supporting Information for the article in journal of Applied Microbiology and Biotechnology

Methanol as a co-substrate with CO_2 enhances butyrate production in microbial electrosynthesis

Hui Yao[a]; Johanna Rinta-Kanto[a]; Igor Vassilev[a]; Marika Kokko[a]*

[a] Faculty of Engineering and Natural Sciences, Tampere University, Korkeakoulunkatu 8,33720 Tampere, Finland

* Corresponding author: Tel.: +358 50 4478 751, e-mail: marika.kokko@tuni.fi

Materials and Methods

Two control serum bottle experiments (duplicates) were done to understand the role of supplied electricity/hydrogen in methanol assisted microbial electrosynthesis. 90 mL of the cathodic medium was added to a serum bottle and sparged with N_2 for 30 mins to eliminate oxygen, following by adding 10 mL enriched culture as the inoculum before starting the experiment. Same CO_2 and methanol addition strategies were used in the experiment, i.e., CO_2 was sparged through the serum bottle every two or three days for 15 mins with the flow rate of 0.4 mL min^{-1} and methanol containing medium was added after the CO_2 sparging to reach the methanol concentration of 20 mmol L^{-1} in the serum bottle. Gas bags were connected to the serum bottle to collect any possible gas production. Analysis of the gas and liquid samples was carried out by following the methods in the main text. In total, the experiments were conducted under 35 ℃ for eight days.

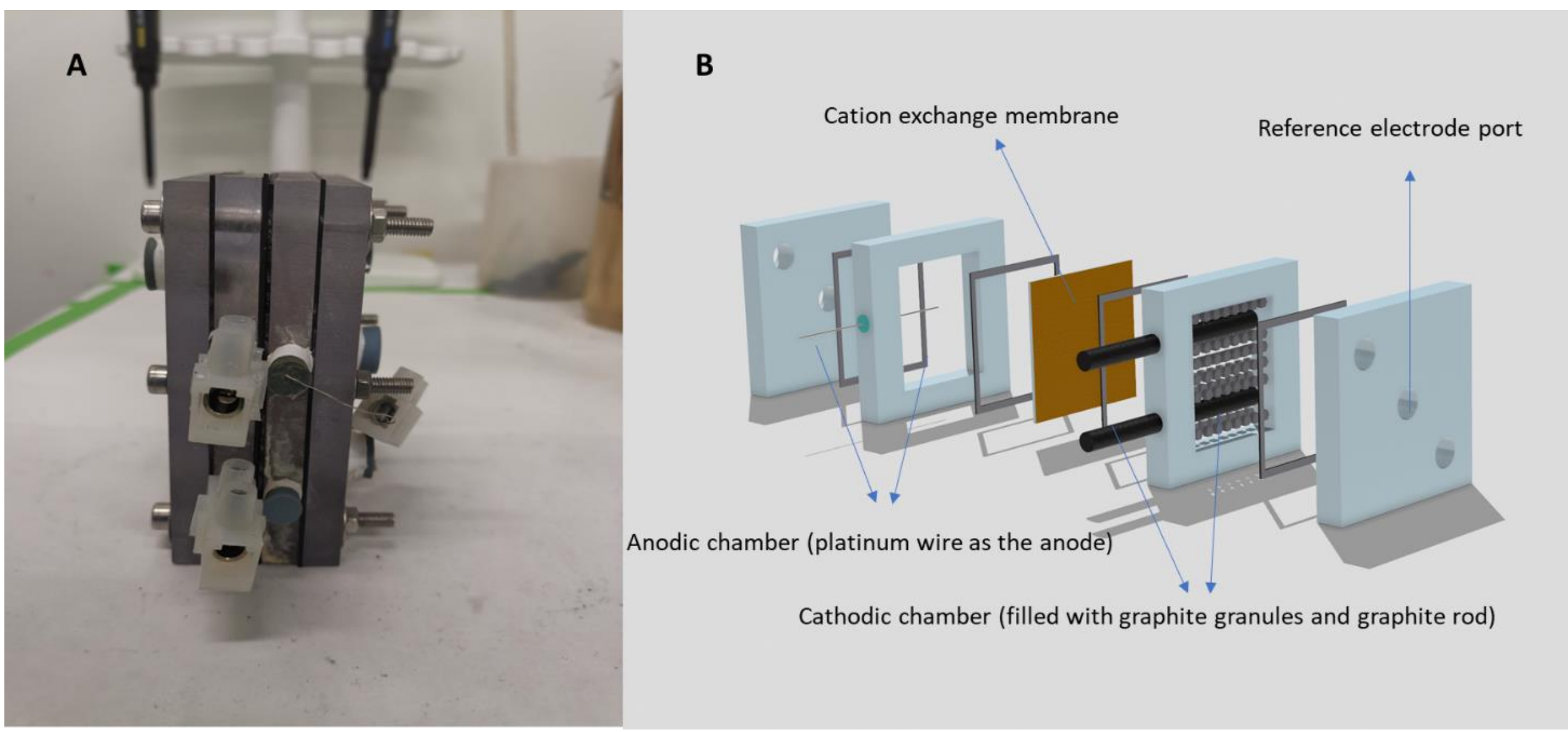

Figure S1 Reactor set-up used in this study. A: Picture of the reactor. B: Schematic illustration of the reactor. The chamber dimensions are 7 cm x 5 cm x 1 cm (internal volume of 35 cm^3). Platinum wire was used as the anode, which was connected through a rubber stopper. Cathodic chamber was filled with graphite granules, where two graphite rods were used as the current collector for cathode. An Ag/AgCl reference electrode was inserted via a glass capillary frit in the reference electrode port. Cation exchange membrane was placed between cathode and anode. Catholyte and anolyte were recirculated by a peristaltic pump through the ports on the end plates.

Results

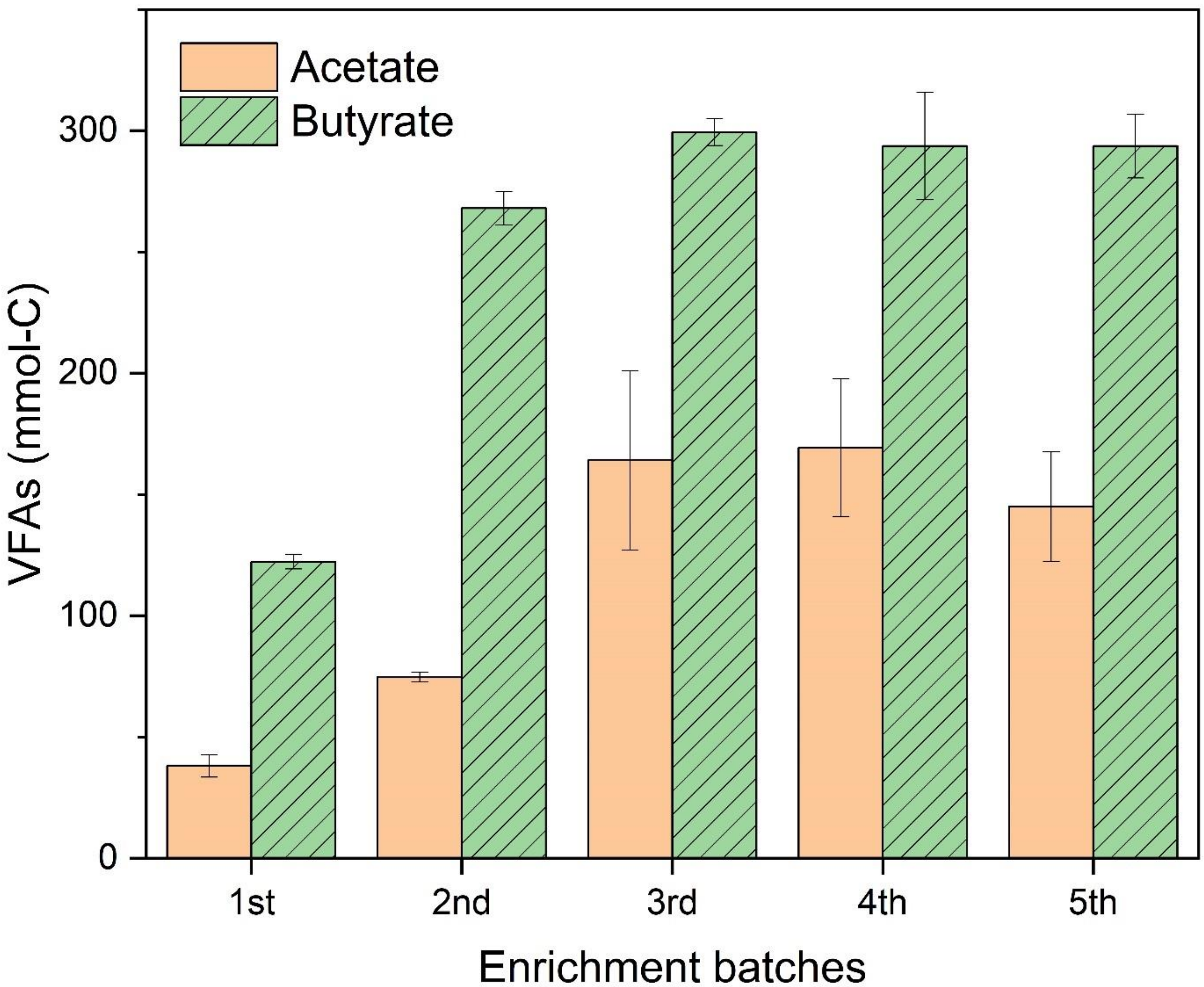

Figure S2. Production of VFAs at the end of each enrichment batch (in g L^{-1}). Error bars indicate the standard deviation in duplicate reactors.

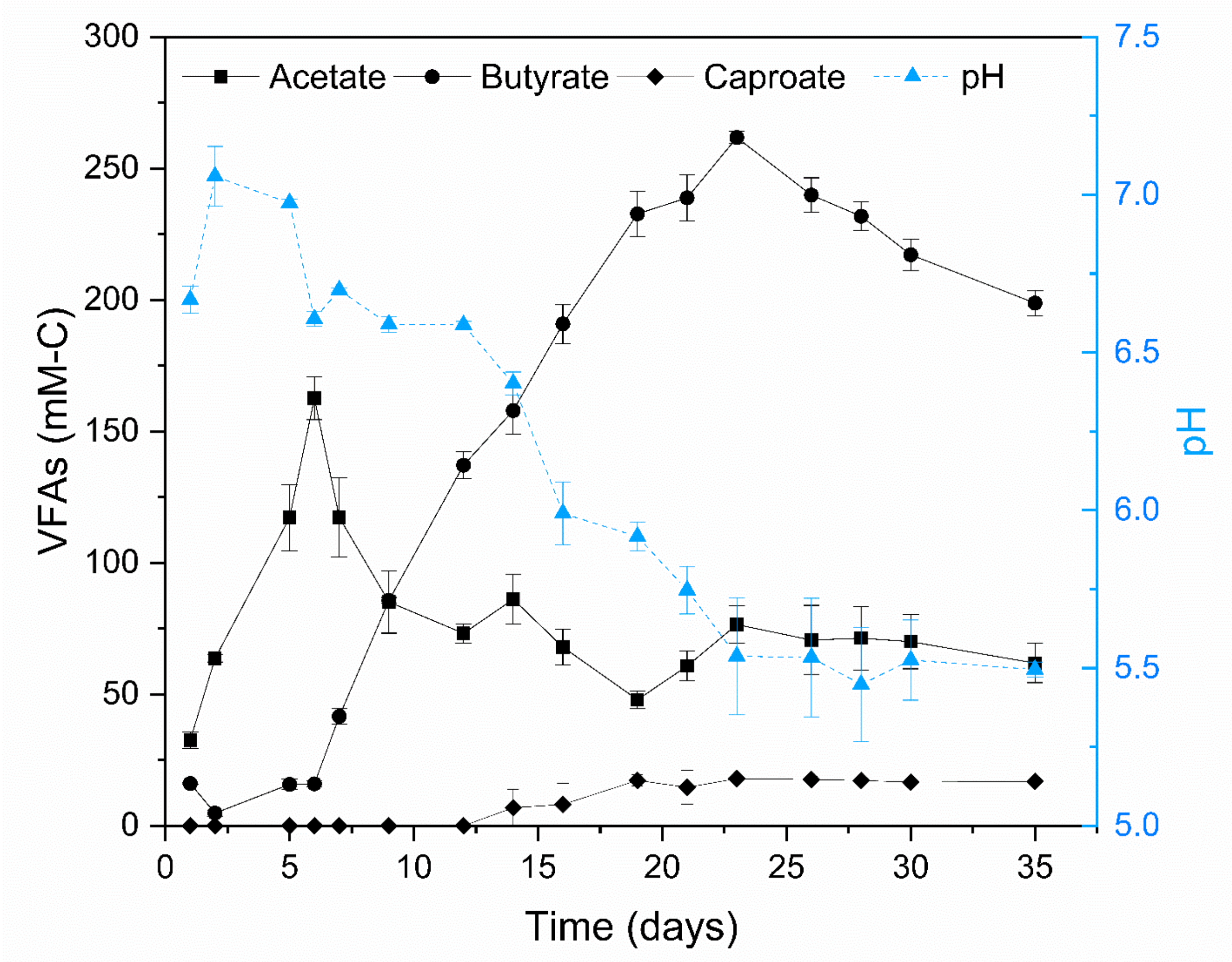

Figure S3. VFA production and pH value of the catholyte from the first enrichment batch. Error bars are the standard deviations between duplicate reactors.

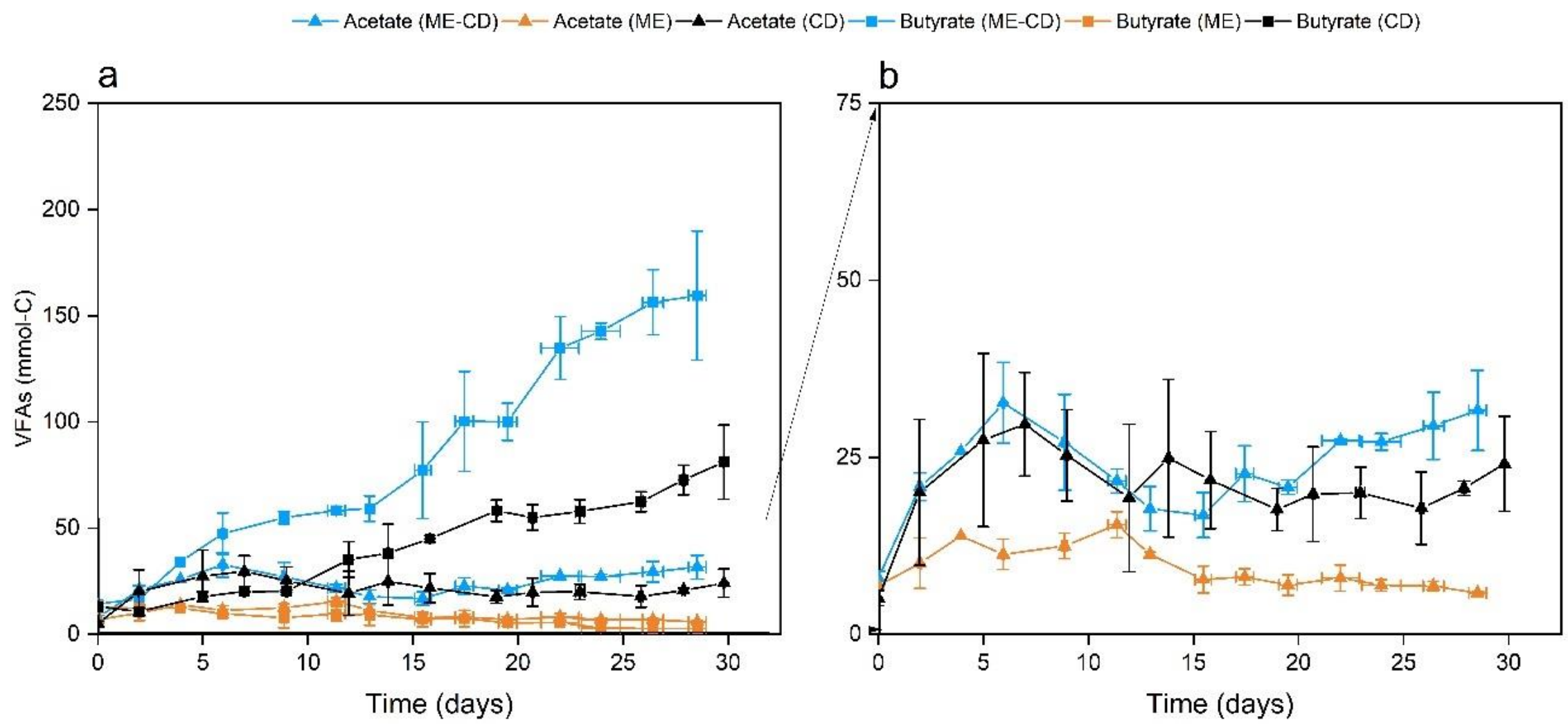

Figure S4. Amounts of VFAs in the co-substrate addition experiments, with a) the overview of all detected VFAs (acetate and butyrate), and b) the acetate production. ME-CD (fed with methanol and CO2); ME (fed with only methanol); CD (fed with only CO2). Error bars are the standard deviations in duplicate reactors.

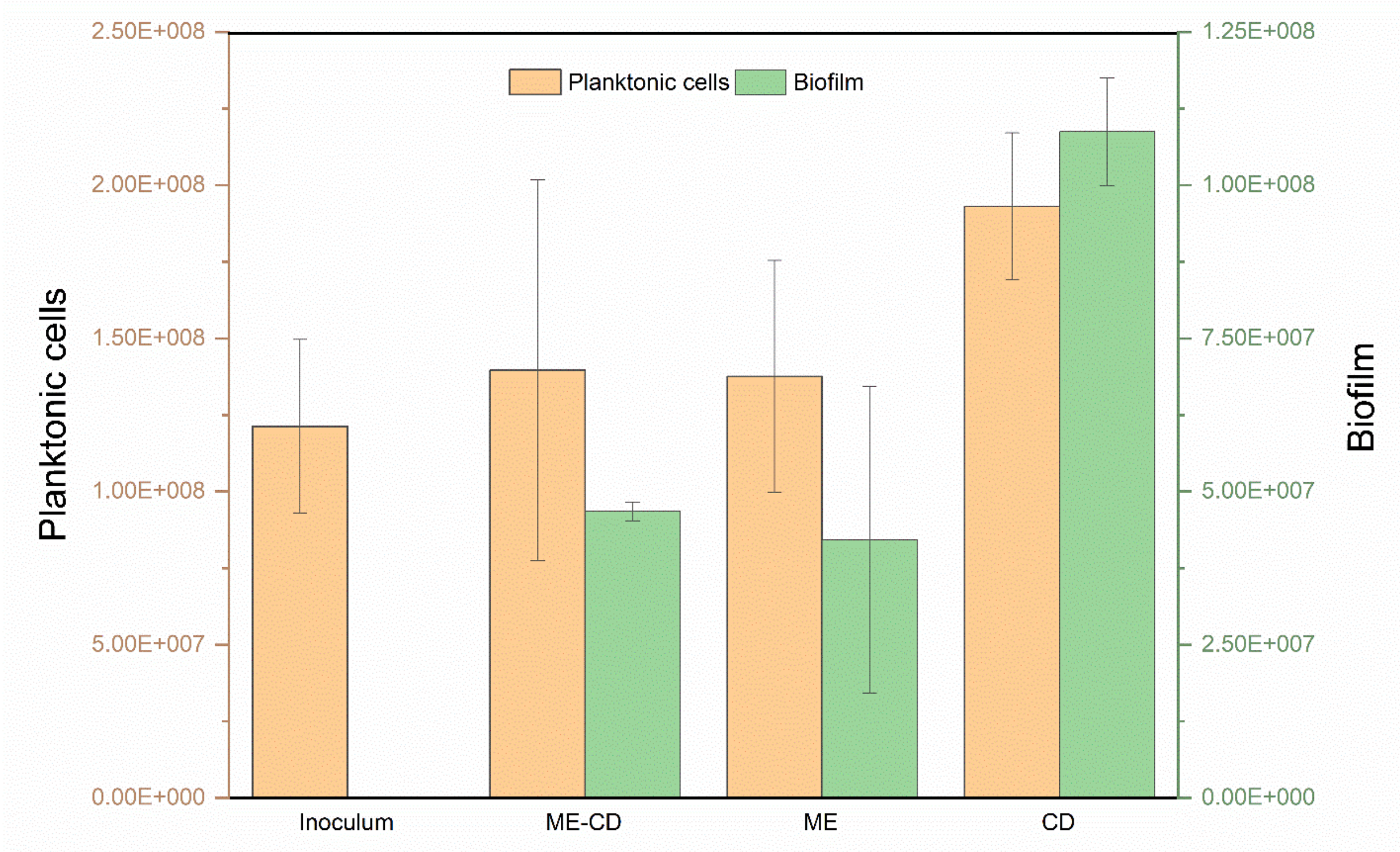

Figure S5. Total 16S rRNA gene copies obtained from the co-substrate addition experiments both for biofilm and planktonic cells from each experimental group. ME-CD (fed with methanol and CO2); ME (fed with only methanol); CD (fed with only CO2). Error bars are the standard deviations between duplicate reactors.

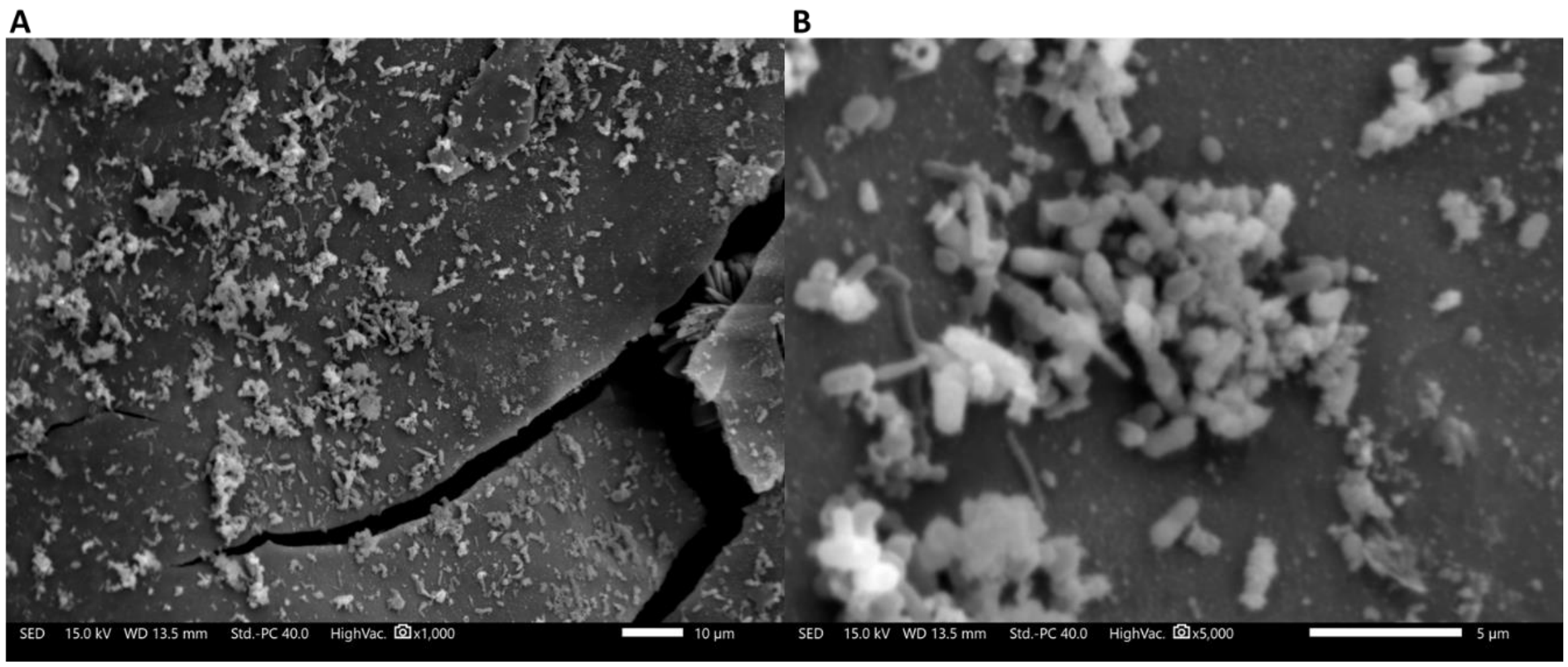

Figure S6. SEM images of the biofilms on the graphite granules from ME-CD reactors. Figure A shows the overview of the surface (1000x). Figure B shows a detailed image of the biofilm (5000x).

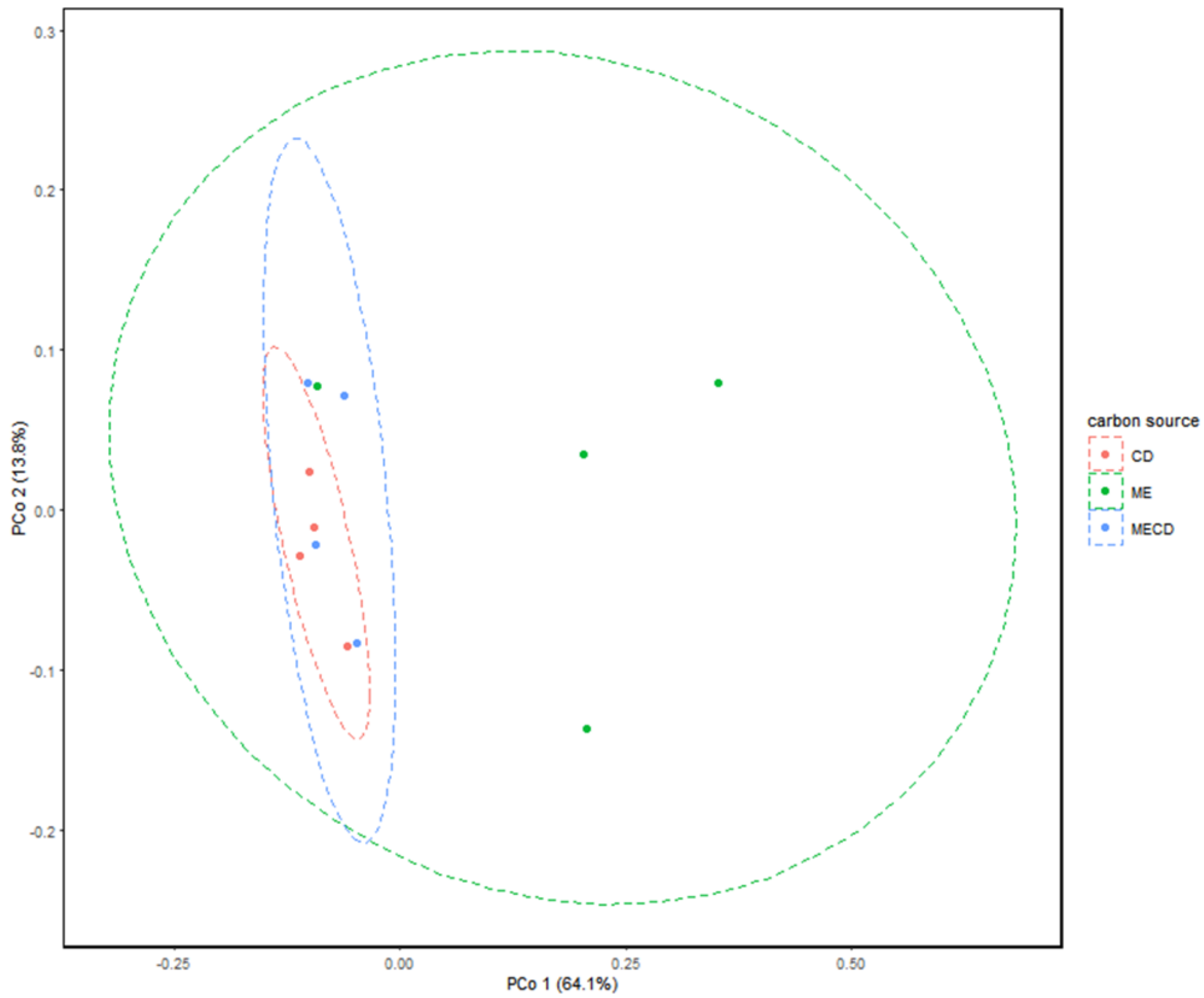

Figure S7. PCoA illustrating the differences in the microbial cultures in three different experimental groups (ME-CD, ME, and CD). Circles indicate the 90% confidential interval. The beta diversity was calculated based on the weighted-UniFrac distance.

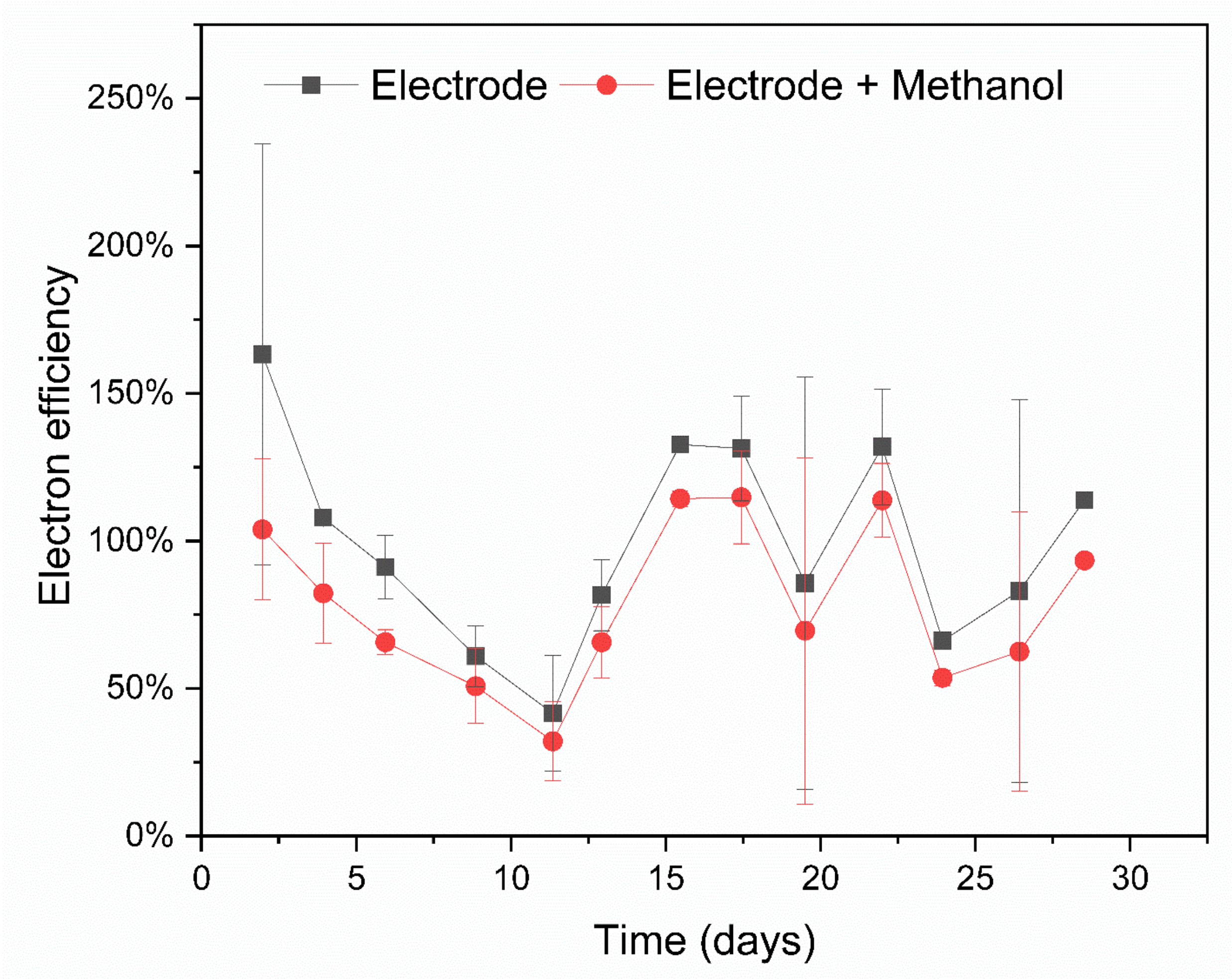

Figure S8. Electron efficiencies between two consecutive sampling timepoints for ME-CD reactors. The electron efficiency was calculated both considering only electrode as the electron source (Electrode) or considering both electrode and methanol as electron sources (Electrode + Methanol). The figure shows that the electron efficiency decreases when considering methanol as an electron source. Most of the electron efficiencies decrease to below 100% indicating the potential role of methanol as an electron source.

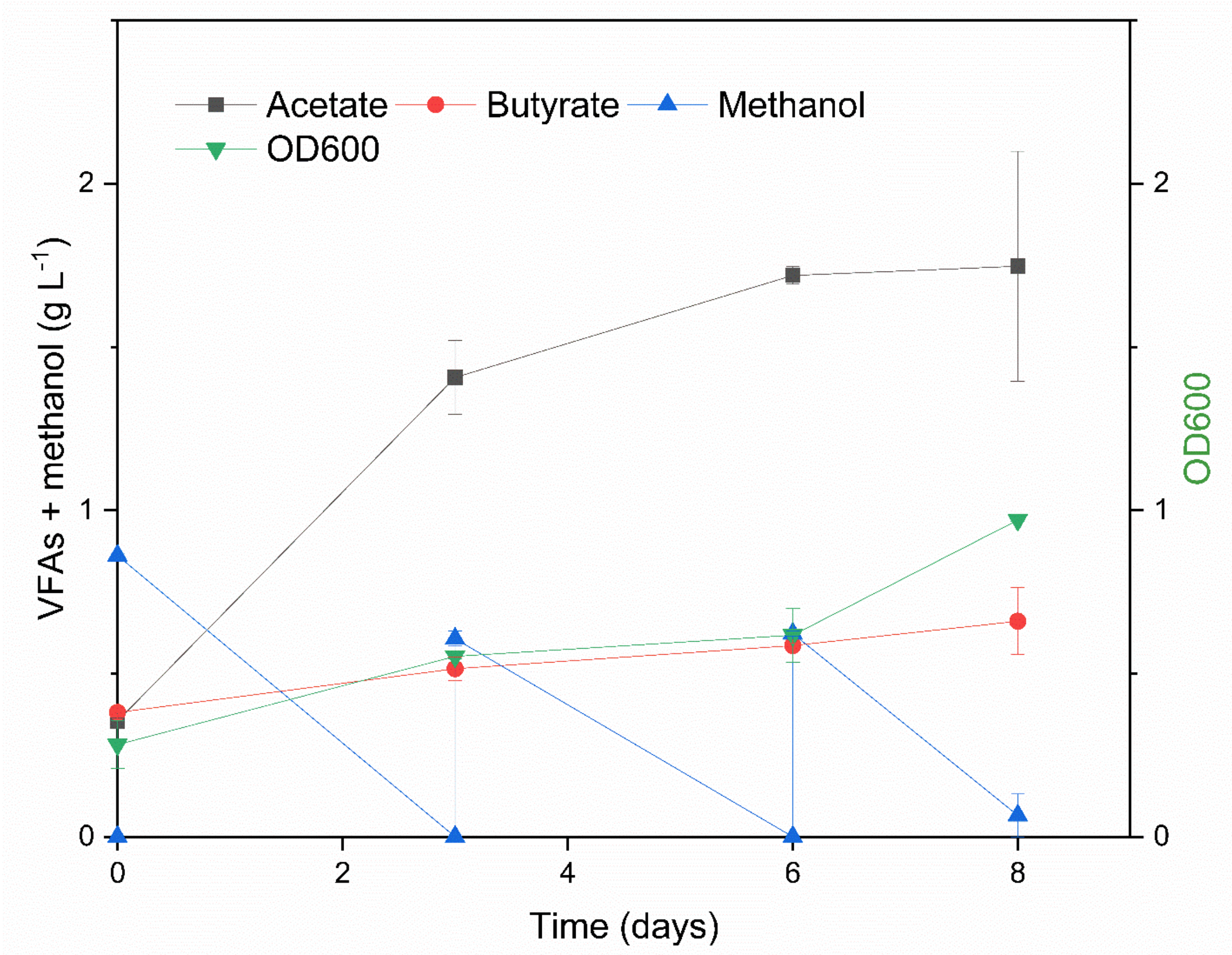

Figure S9. VFAs, methanol concentrations, and OD600 during the control experiments done in batch bottles without electricity and by feeding with CO2 and methanol. Error bars are the standard deviations between duplicate reactors.

Table S1. Composition of the trace elements solution.

Compound	Concentration ($g\ L^{-1}$)
EDTA	10.00
$FeCl_3 \cdot 6H_2O$	1.50
KI	0.18
$CoCl_2 \cdot 6H_2O$	0.15
H_3BO_3	0.15
$MnCl_2 \cdot 4H_2O$	0.12
$ZnSO_4 \cdot 7H_2O$	0.12
$Na_2MoO_4 \cdot 7H_2O$	0.06
$CuSO_4 \cdot 5H_2O$	0.03
$NiCl_2 \cdot 7H_2O$	0.023

Table S2. Composition of the vitamin solution.

Compound	Concentration (mg L-1)
Biotin	2.0
Folic acid	2.0
Pyridoxine-HCl	10.0
Thiamine-HCl	5.0
Riboflavin	5.0
Nicotinic acid	5.0
D-Ca-pantothenate	5.0
Vitamin B12	0.1
p-Aminobenzoic acid	5.0
Lipoic acid	5.0

Table S3. Caproate concentrations at the end of the five enrichment batches of the microbial culture. Standard deviations are obtained from duplicate reactors.

Enrichment batches	Caproate (mM-C)
1st	7.4 ± 4.1
2nd	21.0 ± 5.0
3rd	21.6 ± 1.8
4th	16.9 ± 1.1
5th	28.2 ± 0.8

Table S4. Repeated measures ANOVA for acetate and butyrate concentrations between ME-CD and CD

**ANOVA**					
**Source of Variation**	**SS**	**DF**	**MS**	**F**	**P-VALUE**
Acetate	51.6964443	1	51.69644431	1.058934	**0.312937**
Butyrate	9457.65089	1	9457.650886	104.2338	**1.37E-10**

Table S5. The total inorganic carbon present in the catholyte between feeding timepoints in the co-substrate addition experiment.

	**Total inorganic carbon (mmol)**											
Days	**ME-CD1***		**ME-CD2***		**ME1***		**ME2***		**CD1***		**CD2***	
	**A****	**B****	**A****	**B****	**A****	**B****	**A****	**B****	**A****	**B****	**A****	**B****
0	0.0	16.6	0.0	20.7	0.0	0.5	0.0	1.5	0.0	20.7	0.0	23.5
2	6.2	22.5	0.5	17.0	0.8	3.1	3.3	5.4	0.2	31.2	0.0	29.8
5	0.0	21.8	0.0	11.3	0.0	0.0	0.0	3.9	0.0	20.0	0.4	19.8
7	1.0	18.5	0.0	4.5	0.0	0.0	1.2	4.7	17.8	30.1	0.7	16.1
9	0.8	12.7	0.0	6.6	0.0	0.0	0.0	0.8	0.0	12.5	0.7	21.6
12	0.0	10.8	0.0	5.5	0.0	0.0	2.0	2.8	1.3	14.3	1.0	15.9
14	0.0	4.6	0.0	5.7	0.0	0.2	0.0	1.2	0.5	12.2	0.1	12.8
16	0.2	7.3	0.0	6.3	0.0	0.0	0.4	1.5	0.0	10.0	0.0	10.8
19	0.0	8.2	0.3	7.4	0.1	0.9	2.1	3.0	0.0	10.3	0.8	13.3
21	0.0	6.9	0.2	6.0	0.1	1.6	0.0	0.2	1.8	27.5	0.8	11.5
23	0.0	8.4	0.4	5.0	0.1	0.2	0.0	4.0	0.9	16.5	0.0	16.8
26	0.0	4.6	0.1	8.2	0.3	0.0	0.0	0.0	0.0	14.8	0.0	4.0
28	0.0	N/A	0.0	N/A	0.0	0.1	0.0	0.0	0.8	14.2	6.4	18.4

*: 1 and 2 represent the duplicate reactors from each substrate feeding strategy.

**: A represents the results before substrate feeding, while B represents the results after substrate feeding.

Table S6. Methanol in the catholyte between feeding timepoints in the co-substrate addition experiment.

Days	Methanol (mmol-C)			
	ME-CD1*		ME-CD2*	
	A**	B**	A**	B**
0	0.0	21.6	0.0	6.6
2	18.3	30.1	2.7	6.1
5	12.0	28.8	0.0	6.5
7	10.3	25.1	0.0	5.0
9	11.7	23.6	0.0	8.5
12	12.9	16.7	0.0	5.8
14	8.2	12.9	0.0	5.4
16	7.0	9.7	0.0	4.3
19	6.0	21.7	0.0	6.0
21	8.0	12.3	0.0	4.6
23	3.7	9.7	0.0	4.5
26	0.0	10.3	0.0	5.0
28	0.0	N/A	0.0	N/A

*: 1 and 2 represent the duplicate reactors from each substrate feeding strategy.

**: A represents the results before substrate feeding, while B represents the results after substrate feeding.

Table S7. Methanol concentration during the abiotic experiments

	g L^{-1}		mM	
days	A*	B*	A*	B*
0	**0.0**	**0.62**	**0.0**	**19.4**
2	**0.61**	**1.3**	**19.1**	**39.6**
4	**1.1**	**1.7**	**33.2**	**52.6**
7	**1.5**	**N/A**	**47.0**	**N/A**

*: A represents the results before substrate feeding, while B represents the results after substrate feeding.

Table S8. Result of searching the most abundant OTU in the NCBI 16s ribosomal RNA sequence (Bacteria and Archaea type strains) database using Megablast (September 2022).

**NCBI Reference SEQUENCE:**	**Description**	**Score (Bits)**	**Query cover**	**E Value**	**Max Identify**
NR_026330.1	*Eubacterium callanderi* strain DSM 3662 16S ribosomal RNA, partial sequence	750	100%	0.0	100%
NR_113248.1	*Eubacterium limosum* strain JCM 6421 16S ribosomal RNA, partial sequence	737	100%	0.0	100%
